# BTK inhibition limits microglia-perpetuated CNS inflammation and promotes myelin repair

**DOI:** 10.1007/s00401-024-02730-0

**Published:** 2024-04-24

**Authors:** Anastasia Geladaris, Sebastian Torke, Darius Saberi, Yasemin B. Alankus, Frank Streit, Sabrina Zechel, Christine Stadelmann-Nessler, Andreas Fischer, Ursula Boschert, Darius Häusler, Martin S. Weber

**Affiliations:** 1 Institute of Neuropathology, University Medical Center, Georg August University, Robert-Koch-Str. 40, 37075 Göttingen, Germany; 2 Fraunhofer Institute for Translational Medicine and Pharmacology, Göttingen, Germany; 3 Experimental and Clinical Research Center, Charité - Universitätsmedizin Berlin and Max-Delbrück-Center for Molecular Medicine, Berlin, Germany; 4 EMD Serono, Inc., Billerica, MA USA; 5 Department of Clinical Chemistry, University Medical Center, Göttingen, Germany; 6 Ares Trading SA, Eysins, Switzerland; 7 Merck KGaA, Darmstadt, Germany; 8 Department of Neurology, University Medical Center, Georg August University, Robert-Koch-Str. 40, 37075 Göttingen, Germany

**Keywords:** Multiple sclerosis, Progression, Microglia, BTK inhibition

## Abstract

In multiple sclerosis (MS), persisting disability can occur independent of relapse activity or development of new central nervous system (CNS) inflammatory lesions, termed chronic progression. This process occurs early and it is mostly driven by cells within the CNS. One promising strategy to control progression of MS is the inhibition of the enzyme Bruton's tyrosine kinase (BTK), which is centrally involved in the activation of both B cells and myeloid cells, such as macrophages and microglia. The benefit of BTK inhibition by evobrutinib was shown as we observed reduced pro-inflammatory activation of microglia when treating chronic experimental autoimmune encephalomyelitis (EAE) or following the adoptive transfer of activated T cells. Additionally, in a model of toxic demyelination, evobrutinib-mediated BTK inhibition promoted the clearance of myelin debris by microglia, leading to an accelerated remyelination. These findings highlight that BTK inhibition has the potential to counteract underlying chronic progression of MS.

**Supplementary Information:**

The online version contains supplementary material available at 10.1007/s00401-024-02730-0.

## Introduction

Multiple sclerosis (MS) is a demyelinating disease in which inflammatory processes damage the myelin sheaths within the central nervous system (CNS). MS is thought to be mainly driven by infiltrating immune cells [16, 27, 31] and thus, clinical intervention strategies have often focused on managing the activation, infiltration, and effector functions of peripheral immune cells [6, 14]. While these therapies have shown to reduce the frequency of acute MS relapses, continuous deterioration independent of relapses termed chronic progression remains a treatment challenge [4, 22, 26]. Importantly, the parenchymal disintegration leading to progression is not limited to particular disease stages or forms such as secondary- or primary-progressive MS but rather occurs as an MS-intrinsic process early on in the disease [21]. Since progression is thought to be mainly driven by mechanism occurring within the CNS itself, novel strategies, not only targeting peripheral immune cells are urgently needed. The current view is that progression-driving CNS-inflammation is centrally maintained by microglia, the resident immune cells of the CNS. Thus, therapeutic targeting of chronically activated microglia may be particularly desirable [11, 16, 25, 37] and inhibition of the enzyme Bruton´s tyrosine kinase (BTK) may be one promising strategy to do so. BTK is centrally involved in various immune-receptor pathways (for example downstream of the B cell receptor, Fc receptor, toll-like receptor) and mediates the activation and function of B cells, macrophages, and microglia [7, 34, 35]. Accordingly, therapeutic inhibition of BTK provides a dual mode of action, targeting both innate and adaptive immune processes. In principle, BTK inhibitors are small molecules, which may to some extent be capable of crossing the blood–brain barrier (BBB) further underlining the potential that this class of agents promises for the treatment of MS progression.

Here, we provide compelling evidence of the efficacy of the BTK inhibitor, evobrutinib, in limiting microglia-mediated inflammation in vitro as well as in multiple animal models of MS. We observed reduced activation of microglia when treating chronic experimental autoimmune encephalomyelitis (EAE) or following the adoptive transfer of activated T cells into BTK inhibitor-treated animals, a condition in which the CNS-intrinsic reactivation of T cells by microglia is central. Additionally, in a model of toxic demyelination, in which the immune system is model-intrinsically not involved, BTK inhibition promoted the clearance of myelin debris by microglia, leading to an accelerated remyelination. Overall, this study highlights that evobrutinib and potentially the BTK inhibitor class is not only capable of limiting the activation of peripheral immune cells but that they possess the potential to control microglia-mediated CNS-intrinsic inflammation and promote the recovery of CNS function. Thereby, this study provides vital pre-clinical data that will likely support the prospective findings from ongoing or future clinical trials investigating therapeutic BTK inhibition by evobrutinib and other small molecule BTK inhibitors for the treatment of mechanisms driving MS disease progression.

## Results

### BTK is highly expressed in microglia and upregulated in chronic EAE and MS tissue

**Fig. 1:**
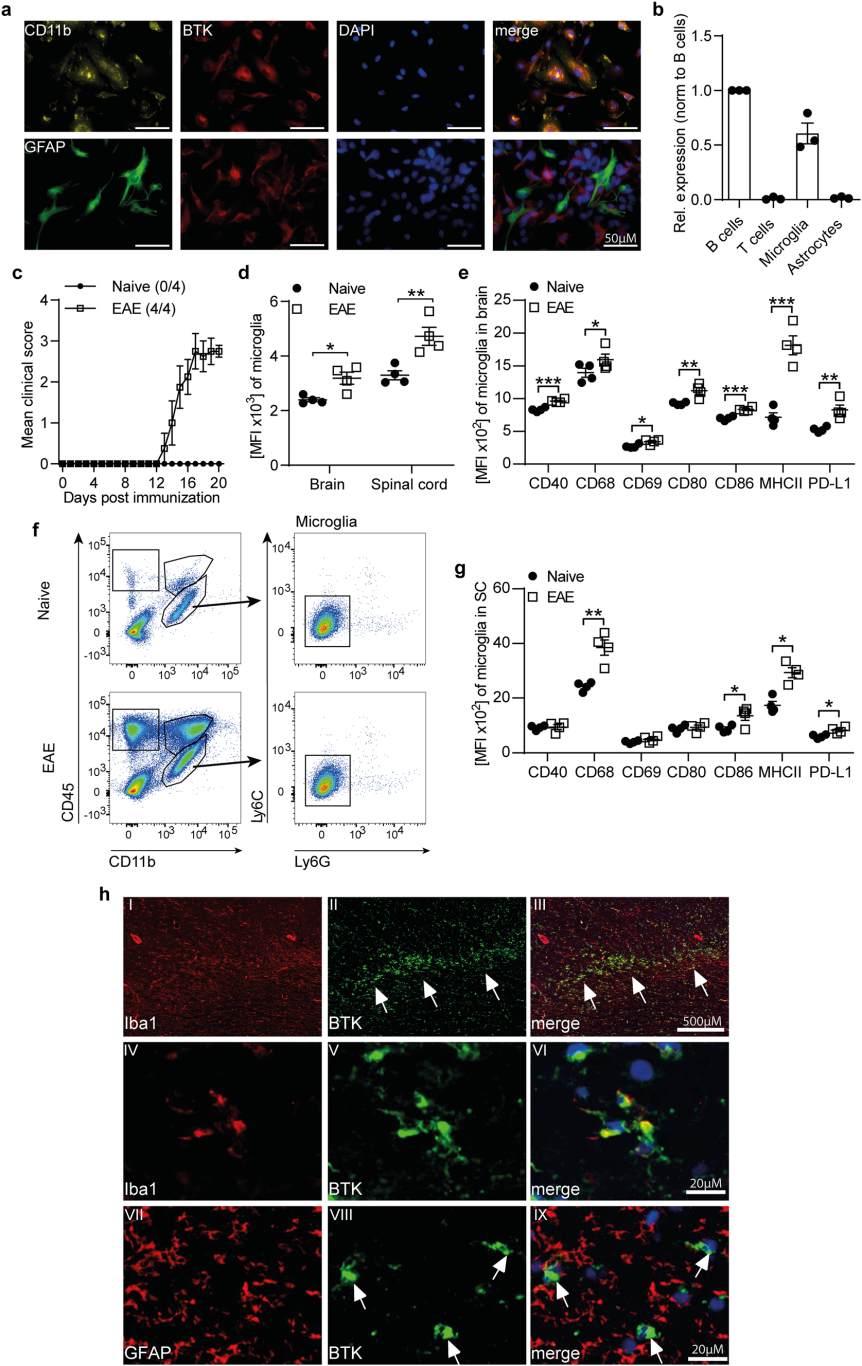
BTK is expressed in microglia and upregulated in CNS inflammation. **a** Primary mixed glia culture isolated from C57BL/6 J were fixed and stained for BTK expression. **b** Splenic B and T cells as well as primary microglia and astrocytes isolated from C57BL/6 J mice were lysed for RNA extraction. mRNA expression of BTK is normalized to GapDH (*n* = 3). **c–g** C57BL/6 J mice were immunized with MOG peptide 35–55. **c** Group EAE score (*n* = 4). **d** Ex vivo BTK expression was analyzed by flow cytometry in microglia isolated from the brain and spinal cord. **e** Microglia were isolated from brain and **g** spinal cord (SC). Changes in the expression of disease-associated microglial markers were analyzed by flow cytometry and are shown as mean fluorescence intensity (MFI, *n* = 4). **f** Gating strategy of microglia isolated from the CNS (CD11b^+^CD45^low^Ly6C^−^Ly6G^−^). **h** Brain biopsy of chronically active (smouldering) MS lesion display BTK expression in Iba1 positive activated microglia (arrow head in I-VI) but not in GFAP positive astrocytes (arrow head in VII-IX). The mean ± standard error of the mean is indicated in all graphs. Data sets are representative of at least two independent experiments. Asterisks indicate significant differences calculated using unpaired two-tailed *t*-test (*P ≤ 0.05, **P ≤ *0.01*, ***P ≤ *0.001*)

To determine if CNS-resident cells are susceptible to therapeutic intervention with BTK inhibitors, we first investigated expression levels of BTK in vitro as well as ex vivo in the most common experimental model of MS. We detected high levels of BTK protein expression **(**Fig. 1a**)** as well as mRNA in primary murine microglia and splenic B cells, while astrocytes and T cells lacked BTK expression **(**Fig. 1a, b). Supporting its involvement in CNS inflammation, microglia isolated from the brain and spinal cord of mice immunized with myelin oligodendrocyte glycoprotein (MOG) peptide (p)35–55 revealed upregulated BTK expression, compared with naive mice (Fig. 1c-e). Such upregulation was also detected in splenic as well as CNS-established lymphocytes, macrophages and neutrophils (Supplementary Fig. 1a-c). In addition, microglia showed an enhanced expression of markers involved in pro-inflammatory activation as well as antigen presentation upon immunization **(**Fig. 1f, g**).**

Moreover, in human tissue from patients with MS, BTK immunoreactivity was easily detectable in chronic active MS lesions and demonstrated a co-localization with Iba-1 positive cells (Fig. 1h and Supplementary Fig. 1f). Although it remains unclear whether these cells are microglia or recruited macrophages, these findings suggest that the expression of BTK plays a central role in chronic CNS inflammation and that BTK inhibition of CNS-resident cells is a highly desirable therapeutic goal.

### Evobrutinib treatment ameliorates clinical severity in active EAE and modulates the activation state of microglia in chronic EAE

**Fig. 2:**
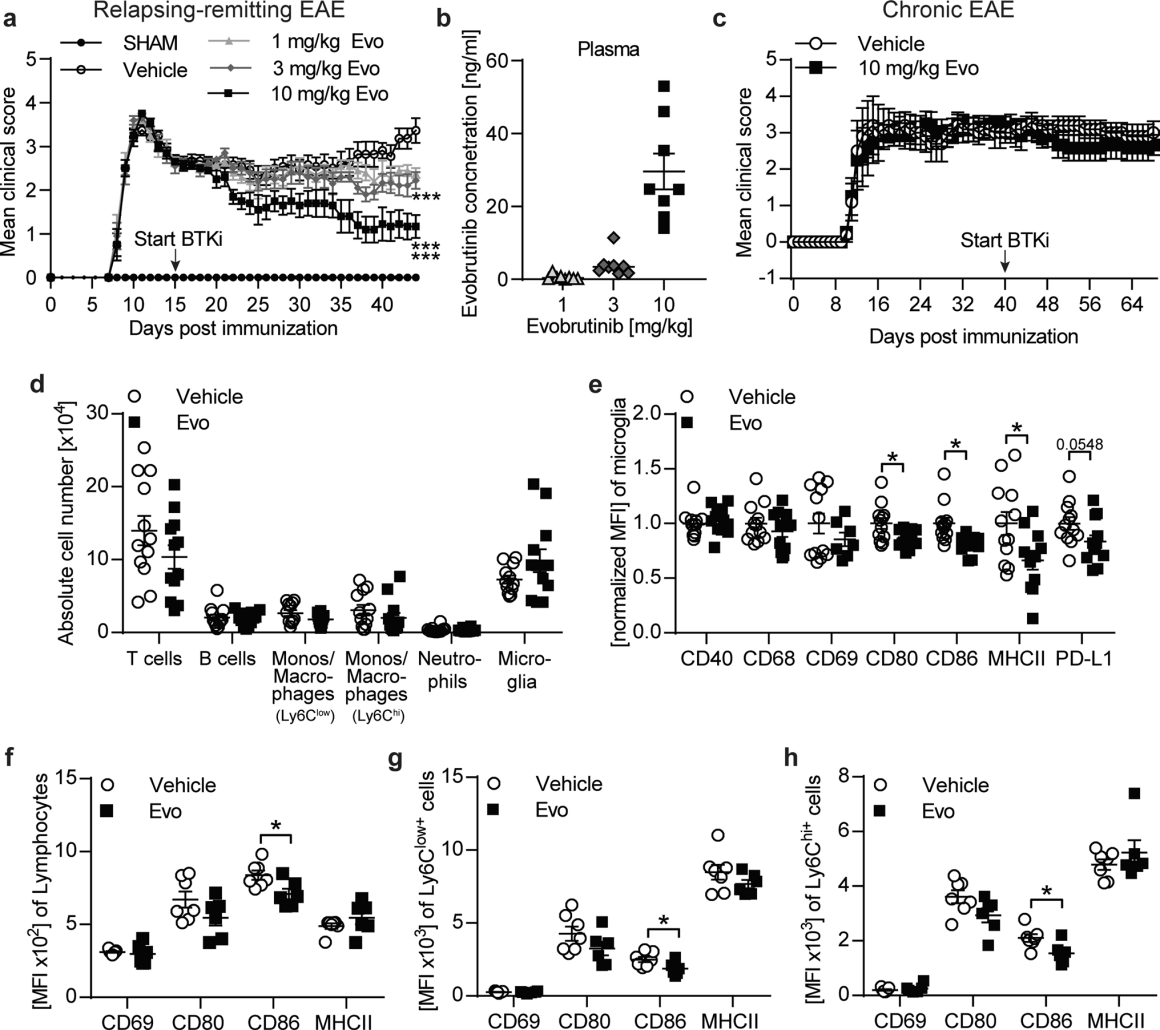
Therapeutic evobrutinib treatment ameliorate disease severity in active EAE and alter microglial phenotype in chronic EAE. **a**, **b** SJL/J mice were immunized with PLP139-151. Treatment with 1, 3 and 10 mg/kg evobrutinib or vehicle control were started at day 15 post immunization and continued daily. **a** Group EAE score (*n* = 10–15). **b** Blood was collected 2 h after treatment and evobrutinib concentration was measured in the plasma (*n* = 10–15). **c–h** C57BL/6 J mice were immunized with MOG peptide 35–55. Treatment with 10 mg/kg evobrutinib or vehicle control were started at the chronic phase of disease at day 40 and continued daily. **c** Group EAE score (*n* = 5–6). **d** Composition of brain-infiltrating and -resident cells (B cells: CD19^+^CD20^+^, T cells: CD3^+^, monos/macrophages: CD11b^+^CD45^hi^Ly6C^low^, monos/macrophages: CD11b^+^CD45^hi^Ly6C^hi^, neutrophils: CD11b^+^CD45^hi^Ly6C^+^Ly6G^+^, microglia: CD11b^+^CD45^low^Ly6C^−^Ly6G^−^) were analyzed by flow cytometry, (*n* = 11–12). **e** Microglia were isolated from the brain. Changes in the expression of disease-associated microglial markers were analysed by flow cytometry and are shown as mean fluorescence intensity, normalized to vehicle control (MFI, *n* = 11–12). **f** Lymphocytes, **g** Ly6C^low+^ macrophages/monocytes and **h** Ly6C^hi+^ macrophages/monocytes were isolated from the brain and changes in expression of markers involved in activation and antigen presentation were analyzed by flow cytometry and are shown as mean fluorescence intensity (MFI, *n* = 11–12). The mean ± standard error of the mean is indicated in all graphs. **a**, **c** Statistical analyses for significant differences on clinical scores over time was performed using Friedman with Dunn’s post-hoc test. **d–h** Data sets are pooled or representative from at least two independent experiments. Asterisks indicate significant differences calculated using the unpaired two-tailed *t*-test (*P ≤ 0.05, **P ≤ *0.01*, ***P ≤ *0.001*)

We have previously shown that evobrutinib decreases clinical severity and pathology in an EAE model with prominent B cell involvement [34]. To further investigate the potential of BTK inhibition in a model in which B cells do not contribute in a pathogenic manner [5], we analyzed the effect of BTK inhibition in an active PLP139-151 induced EAE. Therapeutic treatment with the BTK inhibitor evobrutinib dose-dependently ameliorated clinical disease severity, with the most prominent effect with 10 mg/kg evobrutinib **(**Fig. 2a**).** We were able to detect evobrutinib in the plasma in a dose-dependent manner **(**Fig. 2b**)**. These data show that the clinical efficacy of evobrutinib correlates with the level found in the plasma and indicates that efficacy is mediated by peripheral or central effects beyond B cells.

To investigate the possible effect within the CNS and the ability of evobrutinib to modulate the activation state of microglia, we utilized a model of chronic EAE. For this, we immunized mice and started with a daily treatment of evobrutinib or vehicle control at the chronic manifestation on day 40 post-immunization, when cellular influx is no longer detectable. While clinical disease severity **(**Fig. 2c**)**, the number of infiltrating cells, and the number of microglia remained unchanged upon BTK inhibition **(**Fig. 2d**),** microglia isolated from the CNS of evobrutinib-treated animals revealed reduced expression levels of markers involved in pro-inflammatory activation and antigen presentation **(**Fig. 2e**).** Of note, infiltrating cells, for example, lymphocytes and macrophages isolated from the brain also showed a reduced expression of markers involved in antigen presentation compared with the vehicle control **(**Fig. 2f-h**)**. These data show, that in a chronic phase of the disease with fully established CNS inflammation, treatment with evobrutinib can dampen microglial activity, presumably by its direct effect within the CNS. However, BTK is also expressed by B cells, macrophages and neutrophils, and a remaining influx of these peripheral cells into the CNS cannot formally be excluded. Therefore, we next investigated the effect of evobrutinib in a passive EAE model to exclude this possibility.

### In vivo evobrutinib pre-conditioned microglia are less prone to inflammatory activation, which limits the extent of persisting disability

**Fig. 3:**
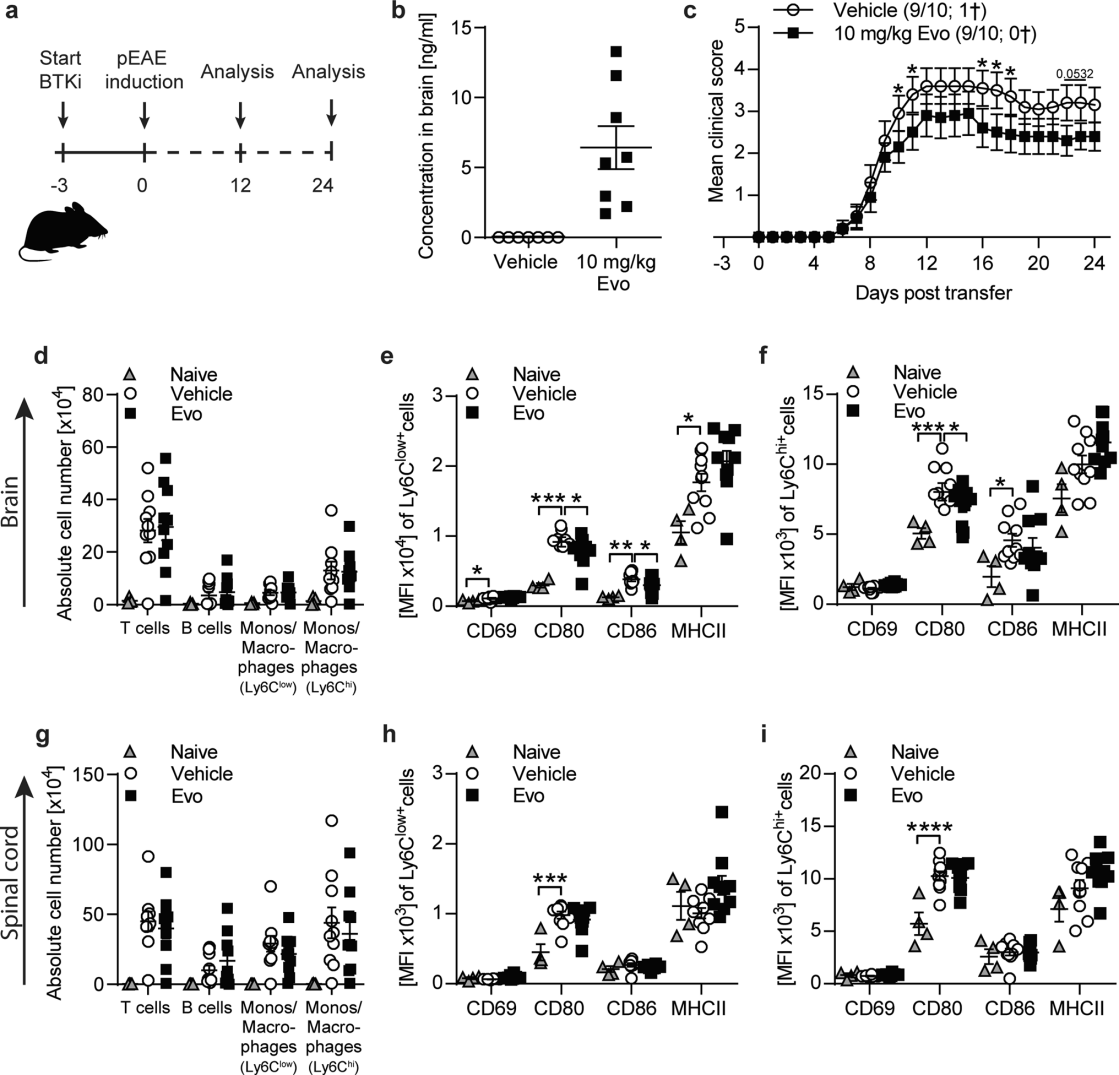
In passive EAE evobrutinib downregulates antigen presenting capacity of CNS monocytes/macrophages. C57BL/6 J mice were treated daily with 10 mg/kg evobrutinib or vehicle control started 3 days prior to passive EAE induction with pathogenic T cells. **a** Overview of experimental setup. **b** Evobrutinib concentration in brain homogenates isolated from mice 24 days post T cell transfer (*n* = 7–8). Tissue was collected 30 min after final dose **c** Group EAE score (*n* = 9–10). **d**, **g** Composition of brain and spinal cord -infiltrating cells (T cells: CD3^+^, monos/macrophages: CD11b^+^CD45^hi^Ly6C^low^, monos/macrophages: CD11b^+^CD45^hi^Ly6C^hi^) were analyzed by flow cytometry on peak of disease (day 12), (*n* = 8–9). Data are shown as mean fluorescence intensity, (MFI, *n* = 9–10). **e–f, h–i** Ly6C^hi+^ and Ly6C.^low+^ macrophages/monocytes were isolated from brain (**e–f**) and spinal cord (**h–i**) on peak of disease (day 12) and changes in markers involved in activation were analyzed by flow cytometry and are shown as mean fluorescence intensity, (MFI, *n* = 9–10). The mean ± standard error of the mean is indicated in all graphs. Data sets are representative from at least two independent experiments. Asterisks indicate significant differences calculated using **c**) unpaired two-tailed *t*-test (*P ≤ 0.05, **P ≤ *0.01*, ***P ≤ *0.001*, ****P ≤ *0.0001*), **d–i** one-way analysis of variance corrected by Holm-Sidak (*P ≤ 0.05, **P ≤ *0.01*, ***P ≤ *0.001*, ****P ≤ *0.0001*)

We assessed to what extent the orally administered BTK inhibitor evobrutinib is found within the CNS. Upon daily treatment with evobrutinib or vehicle control, we found detectable levels of evobrutinib in brain homogenates after 14–27 days of treatment (Fig. 3b and Supplementary Fig. 2b, e), proving that evobrutinib is able to enter the CNS at relevant concentrations. To evaluate to what extent these molecular levels functionally affect the CNS and potentially microglial properties, we investigated the levels of relative BTK occupancy in the acute EAE model by studying the evobrutinib pharmacokinetics/pharmacodynamics (PK/PD) relationship. Evobrutinib showed clinical efficacy in the acute EAE model (Supplementary Fig. 2a). Furthermore, comparable evobrutinib plasma and brain exposure correlates with high BTK occupancy in plasma and brain (Supplementary Fig. 2b, c).

To study the functional consequences of BTK inhibition of microglia in vivo the passive EAE model is of particular interest since it separates the CNS-intrinsic mechanisms from the induction phase of EAE. Adoptively transferred T cells are peripherally raised and expanded, and the outcome of brain inflammation is not influenced by the activation of peripheral lymphatic tissue [19]. To investigate the effect of evobrutinib within the CNS, we started treatment with evobrutinib or vehicle control 3 days before transfer of encephalitogenic T cells. We analyzed CNS-resident and infiltrating cells at the peak of disease and after its chronic disease manifestation (Fig. 3 and Supplementary Fig. 2). Adoptively transferred T cells showed a high purity and activation before transfer (Supplementary Fig. 2f, g). We controlled for the effect of evobrutinib in the mice receiving the T cells and observed that, as expected, evobrutinib treatment inhibited B cell maturation at the specific conversion from follicular (Fo) II to Fo I B cells (Supplementary Fig. 2h).

**Fig. 4:**
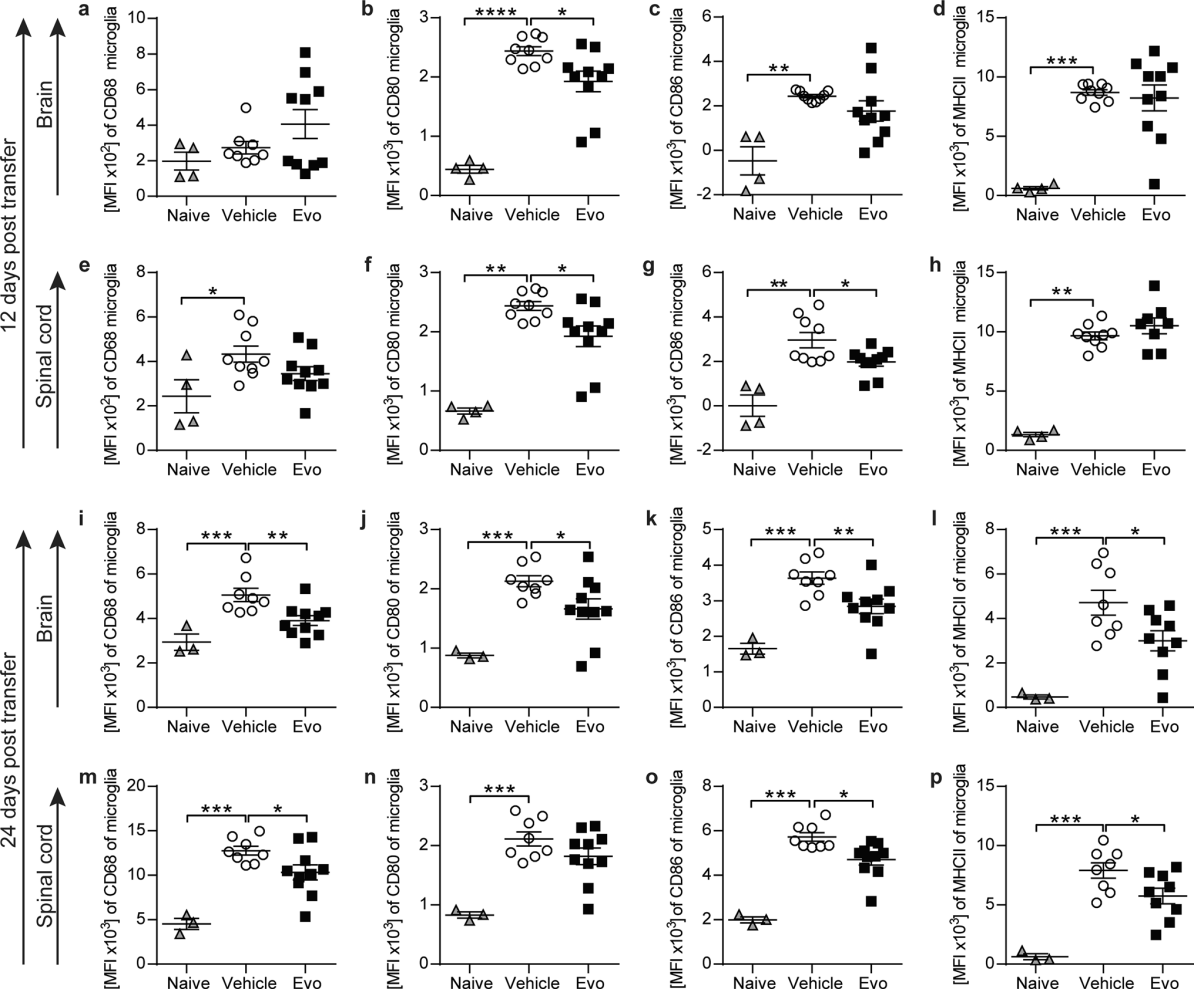
Adoptive transfer of pathogenic T cells leads to a strong microglia phenotype change, which can be dampened by evobrutinib treatment. C57BL/6 J mice were treated daily with 10 mg/kg evobrutinib or vehicle control started 3 days prior to passive EAE induction with pathogenic T cells. **a–d** Microglia were isolated from brain and **e–h** spinal cord on peak of disease (day 12). **i–l** Microglia were isolated from brain and **m-p** spinal cord after peak of disease (day 24). Changes in the expression of markers involved in activation and antigen presentation were analyzed by flow cytometry and are shown as mean fluorescence intensity, (MFI, *n* = *8*–10). The mean ± standard error of the mean is indicated in all graphs. Data sets are representative from at least two independent experiments. Asterisks indicate significant differences calculated using (**a–p**) one-way analysis of variance corrected by Holm-Sidak (*P ≤ 0.05, **P ≤ *0.01*, ***P ≤ *0.001*, ****P ≤ *0.0001*)

Importantly, evobrutinib treatment did not interfere with the induction of EAE by adoptively transferred T cells, as the extent of T cell CNS inflammation was not influenced by BTK inhibition **(**Fig. 3d, g). In contrast, the pro-inflammatory activation state of infiltrating monocytes and macrophages isolated from the brain and spinal cord of recipient mice were reduced in the group treated with evobrutinib (Fig. 3e, f, h, i). Additionally, we observed an evobrutinib-dependent amelioration of the severity of EAE starting at the peak of disease and persisting throughout the EAE course (Fig. 3c). Most importantly, there was a robust EAE-dependent induction in the microglial expression of CD68 in brain and spinal cord, a marker involved in pro-inflammatory function. Furthermore, CD80, CD86 and MHC class II, markers involved in antigen presentation, were increasingly expressed upon EAE induction. At the peak of disease only CD80 expression in the brain and spinal cord as well as CD86 expression in the spinal cord were challengeable by evobrutinib (Fig. 4a-h). Strikingly, the dampening of the expression of molecules mediating pro-inflammatory function by BTK inhibition was strengthened when analyzing microglia at a later stage of disease, as at this stage the expression of all the analyzed surface molecules was reduced (Fig. 4i-p). These data corroborate that BTK inhibition has the ability to directly modulate microglia and CNS-established immune cells.

### Evobrutinib-mediated BTK inhibition dampens pro-inflammatory microglia activation while enhancing phagocytic clearance capacity

**Fig. 5:**
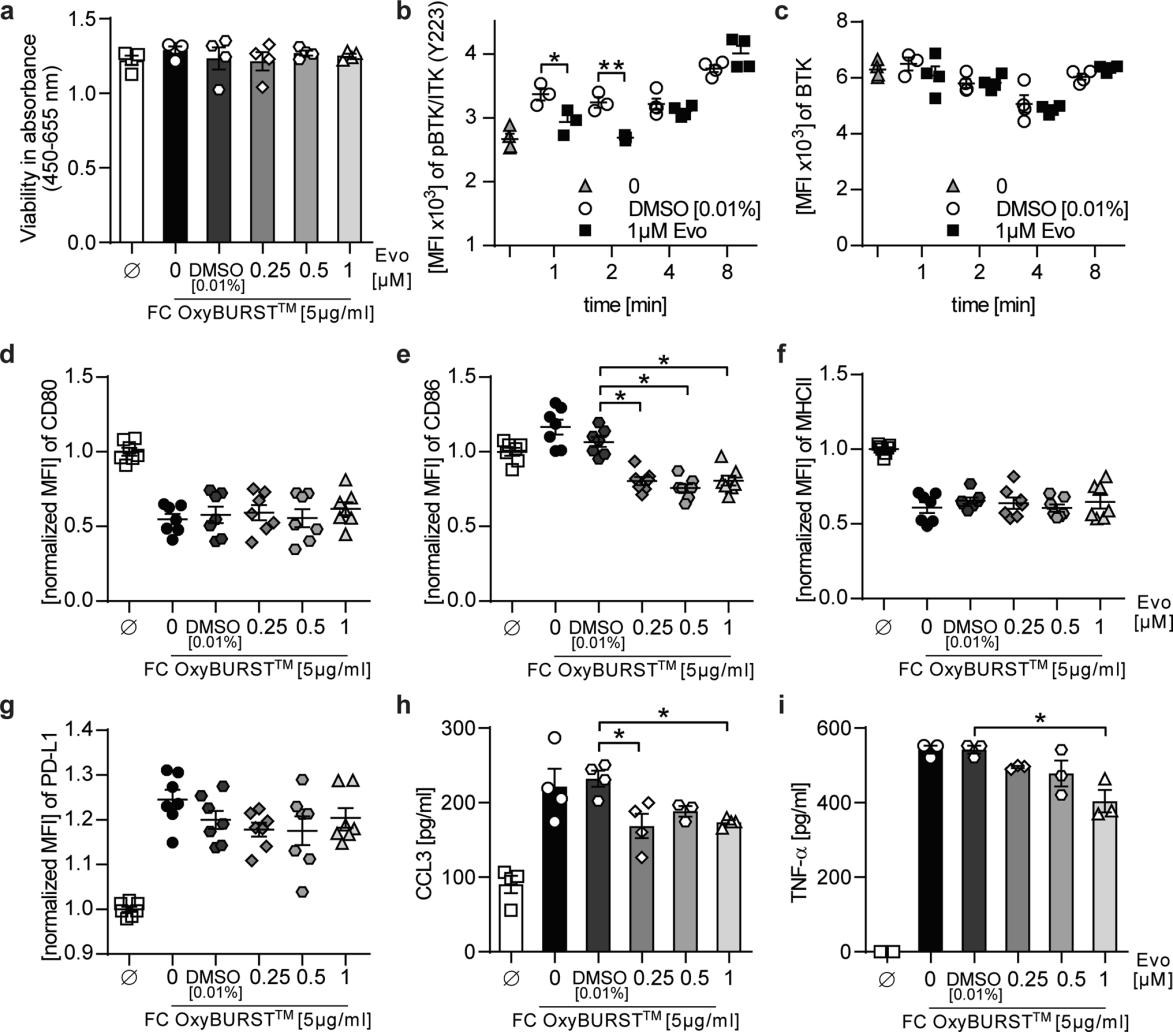
Evobrutinib-mediated BTK inhibition changes disease-associated microglial properties. Primary microglia were either left unstimulated, treated with indicated concentrations of evobrutinib or DMSO control followed by stimulation **a** with 5 µg/ml complexed IgG (FC OxyBurst™) for 18 h. Cell viability was determined using colorimetric WST-1 assay (*n* = 4). **b, c** Stimulated with 5 µg/ml complexed IgG (FC OxyBurst™) for 1, 2, 4, and 8 min. BTK expression and BTK autophosphorylation were analyzed by flow cytometry and are shown as mean fluorescence intensity (MFI, *n* = 4). **d–i** Stimulated with 5 µg/ml complexed IgG (FC OxyBurst.™) for 18 h. **d–g** Changes in the expression of disease-associated microglial markers were analyzed by flow cytometry and normalized to vehicle control and are shown as mean fluorescence intensity, (MFI, *n* = 7, pooled from at least 3 independent experiments). **h, i** Cytokine concentrations were determined by ELISA (*n* = 4 wells/condition. Mean ± standard error of the mean is indicated in all graphs. If not mentioned otherwise, data sets are representative of at least 2–3 independent experiments. Asterisks indicate significant differences calculated using **b–c** unpaired two-tailed *t*-test, **d–i** one-way analysis of variance corrected by Holm-Sidak (*P ≤ 0.05, **P ≤ *0.01*, ***P ≤ *0.001*, ****P ≤ *0.0001*)

These in vivo data revealed a direct effect of BTK inhibition on CNS-resident microglia. To better understand how BTK inhibition exerts these alterations and if the phenotypical changes translate into functional changes, we analyzed BTK inhibition in primary mouse microglia. BTK is involved in the signaling downstream of Fc receptors. To first exclude any toxicity of evobrutinib, we assessed the cell viability of microglia pre-treated with evobrutinib followed by Fc receptor activation and observed no changes in the cell viability (Fig. 5a). To next exclude any off-target effects, we treated primary astrocytes, which do not express BTK (Fig. 1), and observed no alterations upon BTK inhibition (Supplementary Fig. 3i-k).

The activation of BTK results in its autophosphorylation in the kinase domain 223 (Y223). Pre-treatment of microglia with evobrutinib indeed reduced the autophosphorylation of BTK by Fc receptor activation while the overall level of BTK remained unchanged (Fig. 5b, c). We further assessed whether evobrutinib exerts any changes in the Fc receptor-activated microglial phenotype. Microglia showed reduced expression of CD86, a marker which is positively associated with inflammation and antigen presentation [15] (Fig. 5d-g). Furthermore, evobrutinib significantly reduced the production of pro-inflammatory cytokines, such as tumor necrosis factor alpha (TNF-α) and CC-chemokine ligand (CCL)3 (Fig. 5h, i). Besides Fc receptor signaling, BTK is also involved in toll-like receptor (TLR) and various cytokine/chemokine signaling pathways. Therefore, we analyzed microglial phenotype and cytokine production in the presence of BTK inhibition and stimulation of TLR or cytokine signaling. Both conditions revealed an evobrutinib-dependent downregulation of the programed death-ligand 1 (PD-L1), while the expression of other analyzed markers was unaffected (Supplementary Fig. 3a-h).

**Fig. 6:**
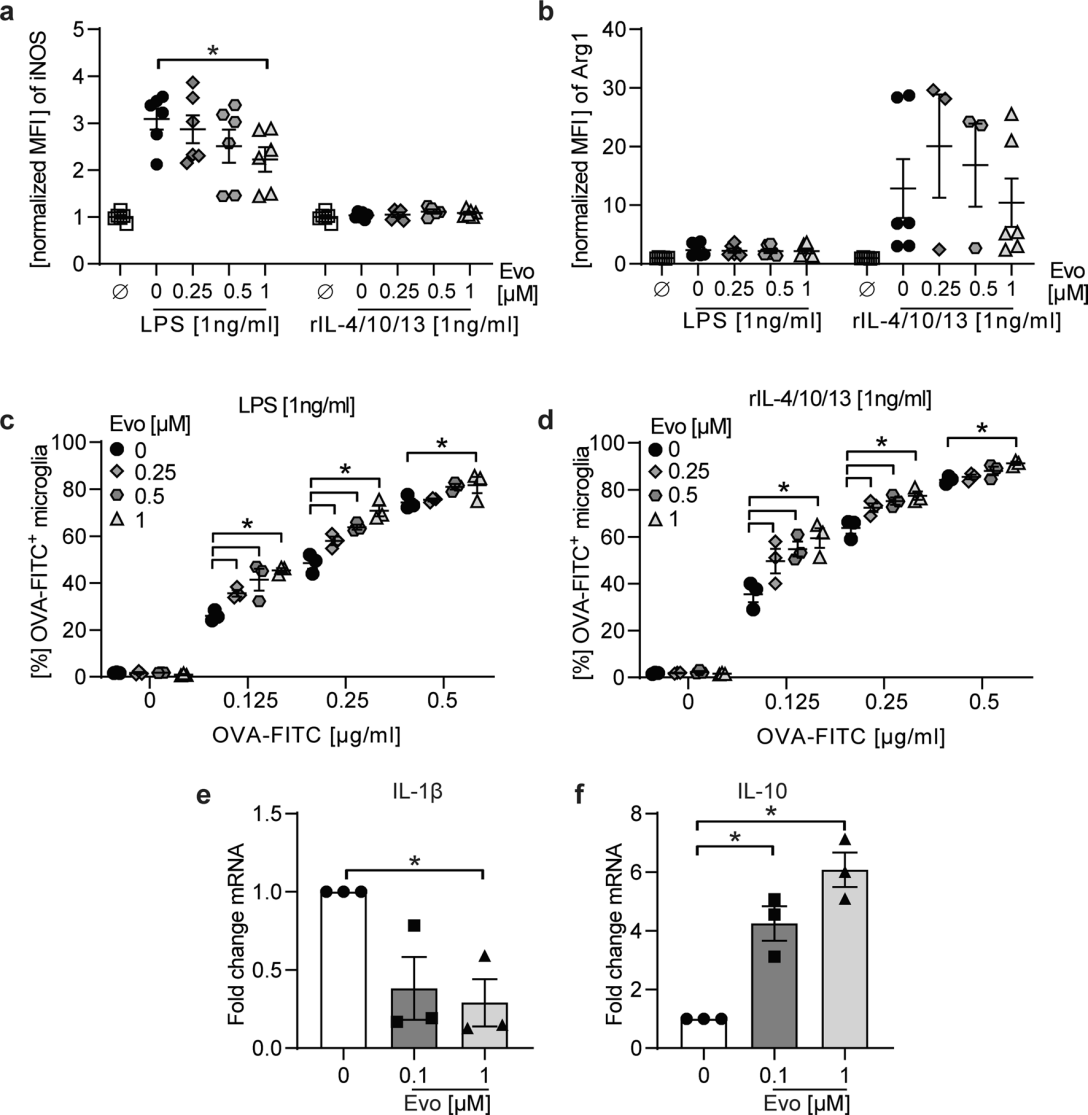
BTK inhibition promotes anti-inflammatory microglia as well as human monocyte phenotype and enhances phagocytosis independent of the stimulatory milieu. **a**, **b** Primary microglia incubated with indicated evobrutinib concentrations followed by culture in the presence of 1 ng/ml LPS or a mix of anti-inflammatory cytokines (recombinant (r) IL-4/10/13) for 18 h. Expression of iNOS and Arginase 1 (Arg1) were analyzed by flow cytometry and normalized to vehicle control and are shown as mean fluorescence intensity, (MFI, *n* = 10, pooled from at least 3 independent experiments). **c**, **d** Following pre-incubation with indicating concentrations of evobrutinib or vehicle and stimulation with LPS or rIL4/10/13, microglia were cultured in the presence of FITC-labelled ovalbumin (OVA-FITC) for 2.5 h and the frequency of phagocytosing OVA-FITC^+^ cells (*n* = 3 wells/condition) was analyzed via flow cytometry. **e**, **f** Primary human monocytes were stimulated with 100 ng/mL GM-CSF and treated with evobrutinib or vehicle for 48 h. Gene expression was measured subsequently by qPCR. The mean ± standard error of the mean is indicated in all graphs. If not mentioned, data sets are representative of at least 2–3 independent experiments. Asterisks indicate significant differences calculated using **a–d** one-way analysis of variance corrected by Holm-Sidak. **e**, **f** Student *t*-test *p < 0.05, compared with GM-CSF control, **g**, **h** Kruskal–Wallis non-parametric test with Dunn ‘s multiple comparison post-test compared to medium control. *P ≤ 0.05, **P ≤ *0.01*, ***P ≤ *0.001*, ****P ≤ *0.0001*

We next assessed whether BTK inhibition may have the ability to also modify microglia plasticity. For this purpose, we used a simplified in vitro approach, in which we analyzed differentiation of primary microglia, in the presence or absence of evobrutinib, following a pro-inflammatory [lipopolysaccharide (LPS)] or anti-inflammatory (recombinant interleukin-4/10/13) stimulation. We measured the competitive enzymes inducible nitric oxide synthase (iNOS) and Arginase-1 (Arg1). Both enzymes convert arginine to either NO or ornithine, respectively and products of each reaction inhibit the opposing reaction. While the enzyme iNOS is upregulated in response to inflammation, Arg1 is upregulated under anti-inflammatory conditions. In the presence of BTK inhibition, we observed a downregulation of iNOS, when stimulated with LPS, whereas the production of Arg-1 remained unaltered (Fig. 6a, b). These data suggest that BTK inhibition has the ability to reduce the pro-inflammatory differentiation of microglia.

To elucidate to what extent these phenotypical changes observed translate to functional alterations, we assessed the ability of evobrutinib pre-treated microglia to phagocytose. Strikingly, in both pro-inflammatory and anti-inflammatory stimulated microglia, treatment with evobrutinib resulted in an enhanced phagocytosis capacity (Fig. 6c, d).

To corroborate these findings in human myeloid cells, we next investigated the effect of BTK inhibition on human monocytes/macrophages activation and differentiation. We observed a time dependent increase of BTK autophosphorylation at position Y223 in the kinase domain after stimulation of human monocytes (THP1 cells), which could be fully inhibited by evobrutinib pre-treatment (Supplementary Fig. 4a, b). Moreover, during differentiation of monocytes, the gene expression level of the pro-inflammatory cytokine IL-1β was reduced while the anti-inflammatory cytokine IL-10 was enhanced upon evobrutinib treatment (Fig. 6e, f). Since the presence of the anti-inflammatory microglial/macrophage phenotype and the clearance of myelin debris is well known to promote effective CNS remyelination, we next assessed whether evobrutinib treatment besides dampening inflammation may facilitate myelin repair.

### Evobrutinib treatment promotes CNS remyelination independent of its anti-inflammatory properties

**Fig. 7:**
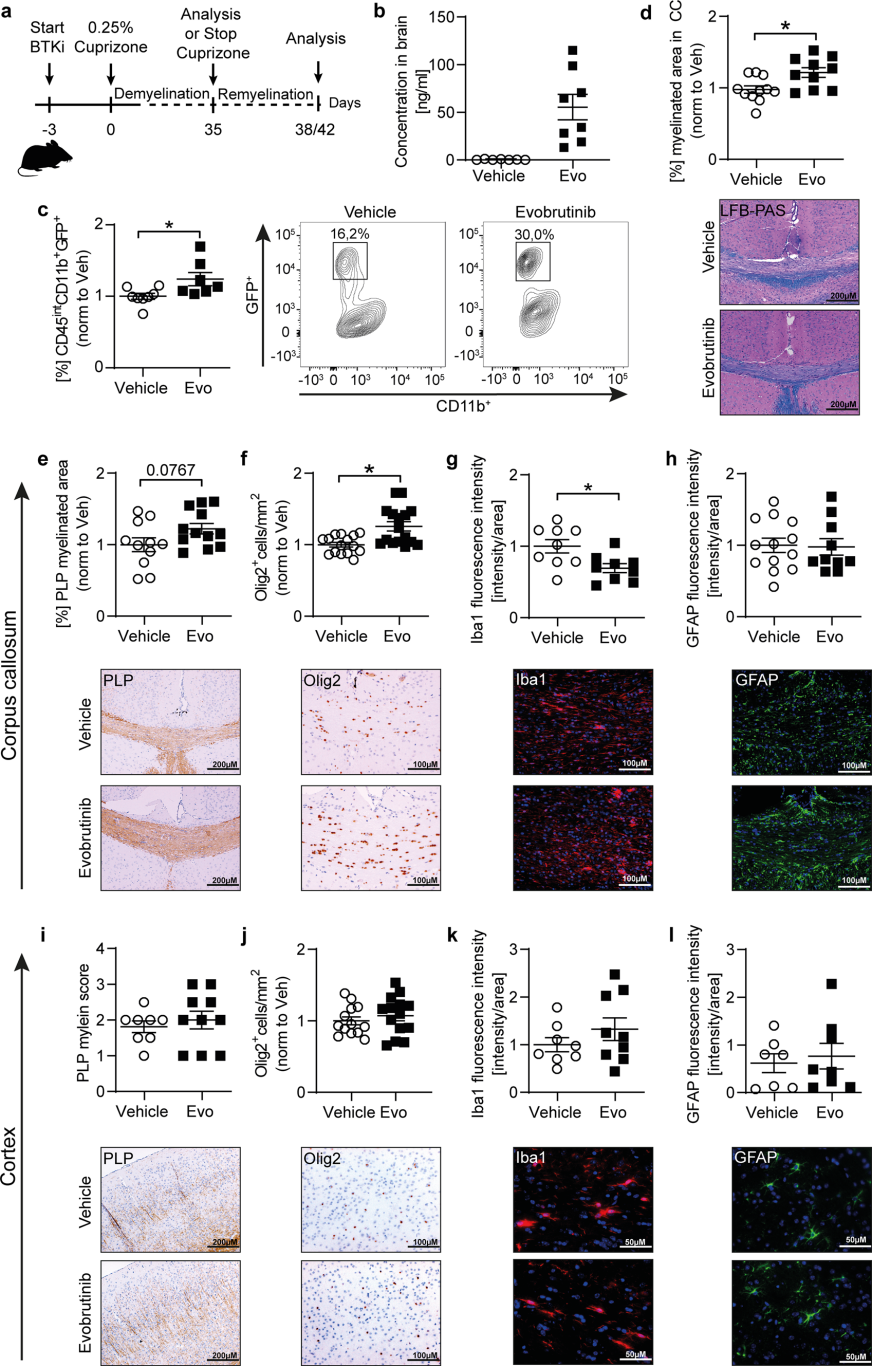
Evobrutinib induces myelin debris clearing in the cuprizone mouse model. **a** Overview of experimental setup. **b** Evobrutinib concentration in brain homogenates from C57BL/6 J mice, treated daily with 10 mg/kg evobrutinib or vehicle control started 3 days prior to simultaneously cuprizone diet for 5 weeks. The tissue was collected 30 min after the final dose. **c** CNP::eGFP transgenic mice were treated daily with 10 mg/kg evobrutinib or vehicle control started 3 days prior to simultaneously cuprizone diet for 5 weeks. Microglia were isolated from the brain and phagocytosis capacity was analyzed by flow cytometry and are shown as mean fluorescence intensity, (MFI, *n* = 7–8). **d–l** C57BL/6 J mice were treated daily with 10 mg/kg evobrutinib or vehicle control started 3 days prior to simultaneously cuprizone diet for 5 weeks followed by 3 days on regular chow. All brain sections were immunohistochemically stained and the corpus callosum (CC) as well as a defined area of the cortex were analyzed. **d**, **e**, **i** Myelinated areas were assessed by **d** luxol fast blue/periodic acid-Schiff (LFB/PAS) staining and **e**, **i** anti-myelin proteolipid protein (PLP) staining and are shown as percentage of myelinated CC in relation to the total CC (**d**, **e**) or scoring of the cortex area (**i**), scale bar: 200 µM. Immunostaining of (**f**, **j**) oligodendrocyte transcription factor 2 (Olig2), number of cells/mm^2^ per group, scale bar: 100 µM. **g**, k Microglia (Iba1) and **h**, **l** astrocytes (GFAP), fluorescence intensity in CC or cortex in relation to the total CC or cortical area, scale bar: 50/100µM. **b–l** Data are shown as mean ± standard error of the mean (SEM). Data are normalized to vehicle and pooled from at least two independent experiments (*n* = 12–16). Asterisks indicate significant difference calculated using the unpaired two-tailed *t*-test (*P ≤ 0.05, **P ≤ 0.01, ***P ≤ 0.001)

**Fig. 8:**
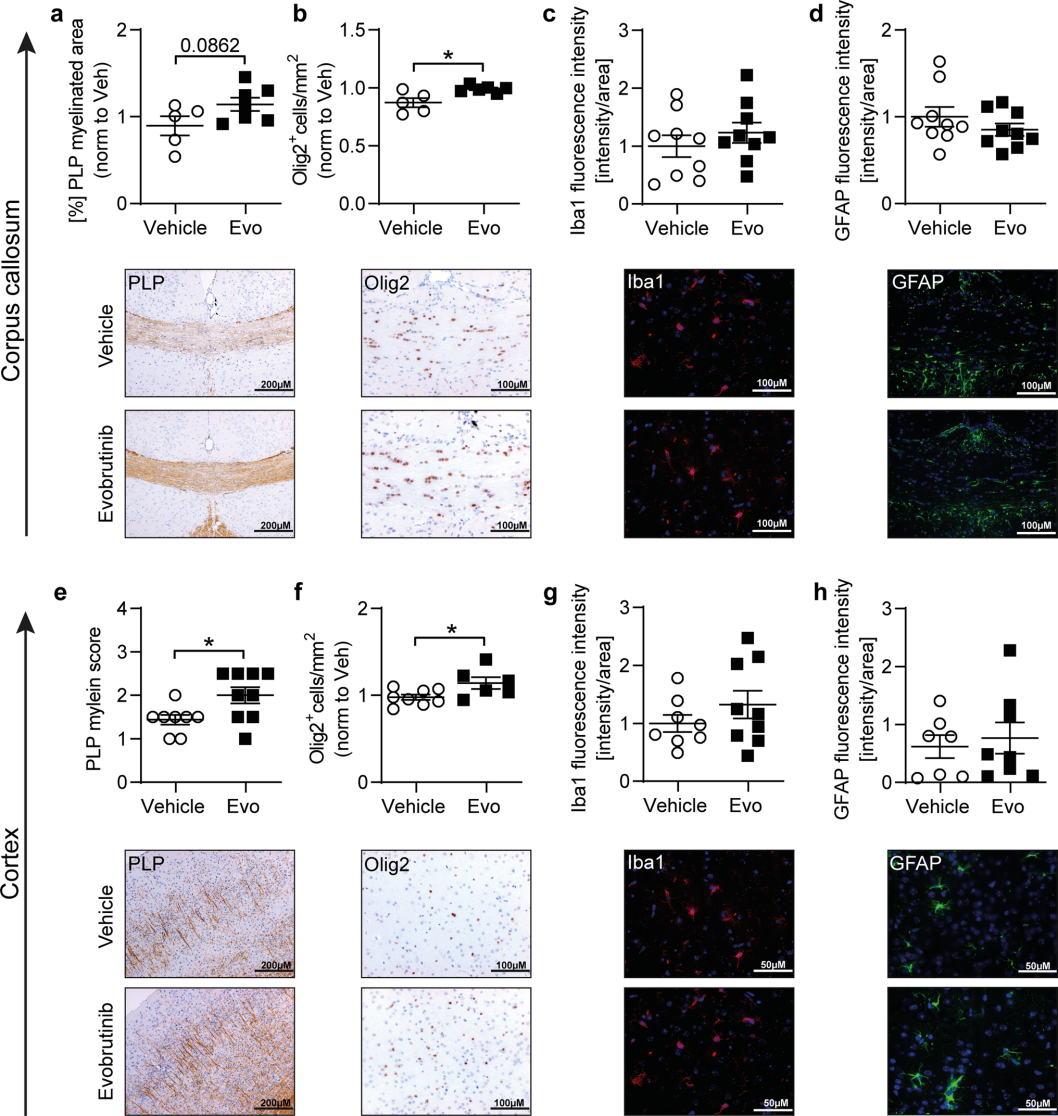
Evobrutinib favors CNS remyelination in the cuprizone mouse model. **a–h** C57BL/6 J mice were treated daily with 10 mg/kg evobrutinib or vehicle control started 3 days prior to simultaneously cuprizone diet for 5 weeks followed by 7 days on regular chow. All brain sections were immunohistochemically stained and the corpus callosum (CC) as well as a defined area of the cortex were analyzed. **a**, **e** Myelinated areas were assessed by anti-myelin proteolipid protein (PLP) and are shown as percentage of myelinated CC in relation to the total CC (**a**) and scoring of the cortex area (**e**), scale bar: 200 µM. Immunostaining of **b**, **f** oligodendrocyte transcription factor 2 (Olig2), number of cells/mm^2^ per group, scale bar: 100 µM. **c, g**, Microglia (Iba1) and **d**, **h** astrocytes (GFAP), fluorescence intensity in CC or cortex in relation to the total CC or cortical area, scale bar: 50/100µM. Data are shown as mean ± standard error of the mean (SEM). Data are normalized to vehicle and pooled from at least two independent experiments (*n* = 12–16). Asterisks indicate significant difference calculated using the unpaired two-tailed *t*-test (*P ≤ 0.05, **P ≤ 0.01, ***P ≤ 0.001)

To investigate whether BTK inhibition enhances myelin clearance and remyelination in vivo, we utilized a toxic model of de- and remyelination. The cuprizone model is used to study processes underlying demyelination and remyelination in a setting not driven by inflammation [39]. Of note, the exchange of the cuprizone diet for the standard diet leads to a rapid endogenous remyelination. We treated mice with evobrutinib 3 days before starting simultaneous treatment with cuprizone for 5 weeks to study demyelination; in another set of experiments, mice were treated with cuprizone for 5 weeks followed by 3 or 7 days of recovery to study early processes underlying remyelination (Fig. 7, 8 and Supplementary Fig. 5). We analyzed the evobrutinib concentration in the brain and found higher levels of evobrutinib in mice exposed to cuprizone when compared with mice from the passive EAE model, probably caused by the prolonged treatment duration (Fig. 7b). During demyelination we observed no histological changes with evobrutinib treatment both in the white (corpus callosum) and grey matter (cortex) (Supplementary Fig. 5). To confirm that evobrutinib promotes microglial phagocytosis, we used the cuprizone model in a transgenic mouse (CNP::GFP), in which oligodendrocytes carry the green fluorescent protein (GFP) [38]. After 5 weeks of simultaneous treatment with evobrutinib and exposure to cuprizone, we isolated microglia from the brain and were indeed able to detect an elevated ratio of microglia positive for GFP, representing microglia that internalized myelin debris in the group treated with evobrutinib **(**Fig. 7c**).** To study whether the enhanced myelin debris clearance may translate into accelerated remyelination, we investigated the corpus callosum (CC) and cortex in the group of mice, which had recovered from the cuprizone diet for 3 days **(**Fig. 7**)** and 7 days **(**Fig. 8**)**. Strikingly, mice treated with evobrutinib showed an enlarged myelinated area in both brain regions (Fig. 7d, e, i and Fig. 8a, e) as well as an elevated number of oligodendrocyte transcription factor (Olig2) positive cells (Fig. 7f, j and Fig. 8b, f). Interestingly, microglial Iba1 fluorescence intensity was decreased after three days of cuprizone withdrawal in the CC, while there was no change observed in the cortex in both timepoints after switching to normal diet (Fig. 7g, k and Fig. 8 c, g). Notably, astrocytes remained unchanged (Figs. 7h, 8). These findings highlight that besides downregulating pro-inflammatory activation and differentiation of microglia, centrally acting BTK inhibition promotes processes of early remyelination, presumably by enhancing clearance of myelin debris.

## Discussion

Here, we first showed that BTK is expressed and functionally upregulated on microglia in chronic CNS inflammation in mice and patients with MS. This is in line with previous studies reporting increased BTK expression levels in MS patients or, more specifically, in active and chronic active MS lesions [8, 12]. Accordingly, microglial BTK is a promising therapeutic target to control chronic CNS-intrinsic inflammation in MS. Secondly, using an in vivo model of MS, we showed that the BTK inhibitor evobrutinib has the capacity to enter the CNS and silence chronic microglia activation. Most importantly, we showed this in three independent models of MS; a) in reversal of acute EAE, b) in late-stage treatment of chronic EAE with a declining influx of peripheral immune cells in the CNS and, most strikingly c) in passive EAE, a setting in which readily primed encephalitogenic T cells directly enter the CNS of evobrutinib-preconditioned naive mice. Thirdly, our in vitro studies revealed that besides dampening activation, evobrutinib prevents microglial differentiation towards a pro-inflammatory phenotype while equipping microglia with an enhanced phagocytic capacity independent on their differentiation. These data suggested that evobrutinib treatment of microglia may promote the clearance of myelin debris, a prerequisite for effective remyelination following acute or chronic inflammatory damage. This we consolidated in the cuprizone model of toxic de- and remyelination; while evobrutinib had no influence on the extent of acute demyelination, we confirmed an evobrutinib-mediated increase in myelin debris clearance by microglia, which in turn lead to an accelerated remyelination.

These findings have several implications: first, evobrutinib, a covalent BTK inhibitor currently trialed for treatment of relapsing MS, is capable of entering the CNS in relevant concentrations, efficiently binding available BTK molecules. Following orally administered evobrutinib for 14–41 days, we measured evobrutinib concentrations in the range of 2.2–114.9 ng/ml in mouse brains, which corresponded to the levels detected in the cerebrospinal fluid (CSF) of patients with relapsing MS (2.27–4.55 ng/ml) who had received evobrutinib 75 mg twice daily for 73 weeks during the open-labeled extension of a Phase 2 trial [30]. Importantly, due to the covalent binding of evobrutinib it is quite plausible that CNS concentrations could rise further with continuous, prolonged treatment [29]. Supporting this notion, as well as the fact that evobrutinib can enter the CNS independent of inflammation, we detected higher levels of evobrutinib (12.9–114.9 ng/ml) in the cuprizone model, most likely due to the fact that the treatment interval time was extended to 38 days. Furthermore, in the acute EAE brain, exposure levels correlated with clinical efficacy and high BTK occupancy in the brain and plasma, supporting target engagement by evobrutinib in the CNS and the periphery. BTK occupancy in peripheral blood mononuclear cells has been used as a surrogate marker for inhibition of BTK activity by first line BTK inhibitors in cancer [2] and clinical data generated during the Phase 2 evobrutinib development program in participants with relapsing MS demonstrated a dose-dependent increase in BTK occupancy in the periphery, with a mean steady state BTK occupancy of > 95% in peripheral blood mononuclear cells [29].

Second, we provide the first direct evidence that the CNS concentrations of evobrutinib achieved by oral administration are capable of dampening chronic microglia activation within the CNS. For this conclusion, the passive EAE setting is key; in this model, entirely naive mice with an intact BBB are treated with evobrutinib prior to adoptive transfer of readily primed encephalitogenic T cells. Therefore, any effects on microglia are achieved prior to EAE induction. Furthermore, to control for any peripheral effects of the BTK-inhibitor treatment, we analyzed B cells within the spleen and did neither observe an activation of B cells by the transfer of T cells, nor an activation-inhibiting effect by the BTK inhibitor treatment. Moreover, T cells themselves do not express BTK, so any direct effect on the donor T cells within the recipient mice can be largely excluded. Transferred T cells are then reactivated within the CNS for example by host microglia, which in our setting have been pre-conditioned with evobrutinib and thus responded much weaker to the inflammatory milieu generated by the infiltrating T cells. Accordingly, these observations strongly suggest that evobrutinib is CNS penetrant independent of ongoing inflammation and functionally capable of down-regulating the activation state of microglia behind a repaired or minorly damaged BBB. These findings provide directly translatable evidence that oral BTK inhibition indeed has the ability to control disease-associated activation of microglia in MS and that evobrutinib or other BTK inhibitors are promising candidates for the therapeutic targeting of perpetuated MS disease activity driven by CNS-intrinsic inflammatory circuits between CNS-resident cells, for example microglia and CNS-established immune cells [10, 18]. Currently, six BTK inhibitors are being tested for the treatment of MS. While all are small molecules, they differ in their distinct characteristics, such as their mode of binding [7, 11]. At present evobrutinib is the only BTK inhibitor to show reduce slowly expanding lesions (SEL) volume in a dose-dependent manner in patients with relapsing MS, with a significant reduction observed with evobrutinib 75 mg twice daily [3]. This is the first evidence that a BTK inhibitor impacts brain lesions associated with chronic inflammation and tissue loss. An integrative, comparative, side-by-side comparison of these BTK inhibitors might be required to assess which is the most promising compound for targeting chronic progression of MS.

Both in primary microglia and human monocytes as well as microglia isolated from naive mice, we observed strong BTK expression which was substantially upregulated upon inflammatory stimulation. Paralleling these observations, BTK was shown to be expressed in human microglia with a pronounced upregulation in human MS [13]. These findings suggest that BTK is involved in the chronic activation of monocytes and microglia in mice and humans, indicating that therapeutic inhibition of BTK may be desirable to control disease-driving activation of monocytes/macrophages and microglia in various forms of MS, including progressive disease. Importantly, EAE and MS lesions are not only composed of microglia but do also contain blood-derived macrophages. Importantly, while the overall clinical benefit is likely mediated by a mixed effect on microglia and macrophages, various experiments can distinguish this further. First, in the chronic EAE setting, we detected a more robust reduction in cellular activation on microglia in comparison to two distinct macrophage subpopulations. Additionally, in the passive EAE setting, there was only a small benefit by BTK inhibition on macrophages in the brain and no detectable effects in the spinal cord (Fig. 3). Lastly, in the cuprizone model, peripheral infiltration is minimal, and macrophages are far outnumbered by resident microglia [24] Nevertheless, in this setting, we can observe that BTK inhibition exerts effects, promoting myelin debris clearance and spontaneous endogenous remyelination by directly influencing microglia.

Aside from this evidence, it may be important to understand how BTK is upregulated in chronic inflammation and, crucially, how BTK is involved in various pro-inflammatory signaling pathways in microglia. Regarding the latter, our in vitro data showed that therapeutic inhibition of BTK counteracts microglia activation by various stimuli, including TLR-ligands and IFN-γ signaling, which is of greatest importance since IFN-γ is largely provided by CNS-infiltrating T cells. These findings are furthermore in line with a study using first line BTK inhibitors, that showed a downregulation of cytokine secretion upon BTK inhibition in TLR-activated BV2 microglia [17]. Strikingly, the inhibitory effect of evobrutinib we observed following Fc receptor stimulation was even more persuasive, which is in line with the established dual mechanism by which BTK is activated, via B cell receptor signaling or Fc receptor signaling [7, 9]. However, the question remains how physiologically or pathophysiologically relevant Fc receptor-mediated activation of microglia is involved in chronic CNS inflammation. In this regard, it has been shown that the basal levels of Fc receptors in microglia are relatively low under physiological conditions, but strongly upregulated in MS lesions [28, 36]. Fc receptors recognize Fc portions of immunoglobulin G (IgG), which was confirmed to promote immune activation of microglia [37]. In patients with MS, oligoclonal IgG is continuously produced and can be detected in the CSF as a diagnostic marker [20, 33]. Accordingly, it is a plausible scenario that clonally expanded, CNS-established plasma cells release IgG, which in turn activates microglia via their Fc receptors and that this constant cellular and molecular interaction promotes and maintains smoldering inflammation within the CNS as the pathophysiological correlate of chronic MS progression. On a related note, we were able to detect an evobrutinib-dependent reduction in the expression of molecules involved in antigen presentation on lymphocytes isolated from the CNS, which from their molecular signature are most plausibly B cells. Therefore, evobrutinib likely has an effect on CNS-established B and plasma cells. Consequently, BTK inhibition as a therapeutic strategy to control underlying mechanisms contributing to MS progression may be particularly effective via its dual actions on microglia and CNS B cells, downregulating microglia activation directly and in addition reducing the production of microglia-activating IgG.

Furthermore, our data suggest that besides its dampening role in neuroinflammation, BTK inhibition is independently able to enhance repair and remyelination in demyelinating diseases, such as MS. In the cuprizone model of toxic de- and remyelination, which develops independently of T and B cell-driven inflammation, we observed an evobrutinib-induced stimulation of microglial phagocytosis associated with and possibly causing accelerated remyelination. This notion is supported by a study using mouse cerebellar slices and in vivo transgenic *Xenopus laevis*, two complementary experimental models of demyelination, in which BTK inhibition was found to favor remyelination [23]. Of note, in our study, there was no effect of evobrutinib on the extent of demyelination, while phagocytosis and myelin repair were enhanced. As one central mechanism leading to the progression of MS is the relative imbalance between damage and repair [1], these repair-promoting properties may be highly desirable from a therapeutic point of view. An enhanced remyelinating capacity mediated by BTK inhibition may promote recovery from acute relapses as well as repair chronic damage in MS. Furthermore, these protective and restoring capacities may be extremely desirable in other demyelinating conditions such as chronic inflammatory demyelinating polyneuropathy or Guillain–Barré syndrome. Our primary observation thus opens a variety of exciting avenues exploring the effects of BTK inhibitors in other diseases and models driven by demyelination.

Taken together, our study uncovers several key findings with implications for the therapeutic positioning of evobrutinib and BTK inhibition as a therapeutic strategy. Besides the well-established modulation of peripheral B cells, orally administered evobrutinib is capable of entering the CNS and of locally limiting the pro-inflammatory activation and differentiation of microglia. Hereby, evobrutinib treatment has the ability to control and dampen inflammatory circuits between chronically activated CNS-resident and CNS-established immune cells, a key pathophysiological mechanism underlying chronic progression of MS. Furthermore, and at least equally exciting, evobrutinib treatment promotes CNS repair by enhancing the clearance capacity of microglia and fostering remyelination. In conclusion, evobrutinib treatment limits the activation of peripheral immune cells, controls and dampens microglia-mediated CNS-intrinsic inflammation and at the same time promotes restoration of CNS function, providing a unique and strong experimental basis for its capacity to halt chronic progression of MS.

## Methods

### Mice

Wild-type C57BL/6 J mice were purchased from Charles River. All animal experiments were carried out in accordance with the guidelines of the Central Department for Animal Experiments, University Medical Center, Göttingen and approved by the Office for Consumer Protection and Food Safety of the State of Lower Saxony (protocol number 33.9-42,502-04-16/2267, 33.9-42,502-04-21/3680, 33.9-42,502-04-17/2745). The SJL mice used in this study were purchased from the Jackson Laboratory of Bar Harbor, ME. All procedures were performed in accordance with the EMD Serono Institutional Animal Care and Use Committee (IACUC). All local and national laws and regulations regarding animal care and use were complied with. The study was conducted according to the global policy “Quality management system (QMS) for Merck Serono Research (MSR)” of Merck KMSC2364447CA (Functional Quality Standard: MSRQMS01QS) and will follow the respective departmental MSR QMS Policy (Department Quality Policy (MSR DQP)).

### Sex as a biological variable

For all active EAE experiments, female mice were examined. For the passive EAE experiments, male and female animals were examined, and similar findings are reported for both sexes. As recipient mice, only female mice were examined. For the cuprizone experiments male and female animals were examined, and similar findings are reported for both sexes.

### Evobrutinib treatment

Evobrutinib was formulated in 20% Kleptose HPB in 50 mM Na-Citrate buffer pH 3.0 and administered at 10 mg/kg body weight (BW) by oral gavage daily.

### Active EAE induction and scoring

Female SJL/J mice were immunized subcutaneously with an emulsification of PLP139-151 (100 mg/mouse) peptide dissolved in PBS and IFA (supplemented with 2 mg/mL Mycobacterium tuberculosis) on day 0 in the abdominal flank, followed by intraperitoneal injections of pertussis toxin (60 ng/mouse) in saline on the day of immunization and 2 days thereafter. Disease severity was scored using a standard scale: 0 = no clinical signs, 1 = limp tail, 2 = impaired righting reflex, 3 = partial hind limb paralysis, 4 = complete hind limb paralysis, 5 = moribund/death (Half scores were used in case the symptoms fell in between two grading’s). If mice reached a score of 4 or more on 3 consecutive days they were euthanized due to humane endpoint. If the mice at any time reached a score above 4, they were euthanized immediately due to humane endpoint.

### Chronic EAE induction and scoring

Female wild-type mice were immunized subcutaneously with 75 µg MOG_35-55_ peptide MEVGWYRSPFSRVVHLYRNGK (Auspep) emulsified in complete Freund’s adjuvant (Sigma-Aldrich) containing 250 µg killed Mycobacterium tuberculosis H37 Ra (BD Bioscience) followed by intraperitoneal injections of 200 ng of Bordetella pertussis toxin (Sigma-Aldrich) on the day of immunization and 2 days, thereafter. EAE severity was assessed daily and scored on a scale from 0 to 5 as follows: 0 = no clinical signs; 1.0 = tail paralysis; 2.0 = hindlimb paresis; 3.0 = severe hindlimb paresis; 4.0 = paralysis of both hindlimbs; 4.5 = hindlimb paralysis and beginning forelimb paresis; and 5.0 = moribund/death.

### Passive EAE

Female wild-type mice were immunized subcutaneously with 200 μg MOG_35–55_ peptide emulsified in Complete Freund’s Adjuvant containing 250 μg killed Mycobacterium tuberculosis (donor mice). After 11–12 days, the primary immunization-draining (inguinal) lymph nodes were isolated and cultivated for 3 days at a density of 2–2.5 × 10^6^ cells in the presence of 20 µg/ml anti-Interferon gamma antibody, 25 ng/ml recombinant Interleukin-12 and 25 µg/ml MOG_35-55_ peptide. Subsequently, the pathogenic T cells were purified by the magnetic-bead associated removal of B cells using the MojoSort CD19 kit (BioLegend). Recipient mice, pre-treated for 3 days with 10 mg/kg BW evobrutinib or vehicle control (Kleptose), received 1.5–2 × 10^6^ T cells intraperitoneally. EAE severity was assessed daily on a scale from 0 to 5 (0 = no clinical signs; 1.0 = tail paralysis; 2.0 = loss of righting reflex; 3.0 = beginning hind limb paresis; 4.0 = paralysis of both hind limbs; 4.5 = beginning forelimb paresis; 5.0 = moribund/death).

### Cuprizone treatment

Demyelination was induced by feeding mice with 0.25% cuprizone (Sigma Aldrich, MO) mixed into standard chow ad libitum for 5 weeks. In an interventional setting, mice were fed with cuprizone for 5 weeks, followed by 3 or 7 days with normal chow.

### Histology and immunohistochemistry

Mice were transcardially perfused with PBS followed by 4% paraformaldehyde (PFA) and tissue was paraffin embedded. One-micrometer thick slices were stained with hematoxylin and eosin and Luxol fast blue/periodic acid shiff (LFB/PAS). CNS tissue was further evaluated by immunohistochemistry with an avidin–biotin technique using antibodies specific for PLP (1:500; Bio-Rad) and Olig2 (1:300; IBL). Histological sections were captured using a digital camera (DP71; Olympus Europa) mounted on a light microscope (BX51; Olympus Europa). The percentage of demyelinated white matter was calculated using cellSens Dimension software (Olympus Europa). The demyelination of the grey matter (cortical area) stained for PLP was scored blinded using a light microscope with a magnification of × 200. The different grades of myelinated areas were assessed using a scale from 0 (complete myelin) to 4 (complete demyelination) as described previously [32]. Oligodendrocytes were quantified at × 400 magnification using an ocular counting grid and are shown as cells/mm^2^. Microglia and astrocytes were detected by fluorescence immunofluorescence. Therefore, slices were pre-treated either with citrate buffer and stained with the primary antibodies anti-Iba1 (1:500; Fujufilm, Wako) and GFAP (1:300; Synaptic System) followed by staining with the respective secondary antibodies anti-rabbit Cy3 (Jackson ImmunoResearch) and 4′,6-diamidino-2-phenylindole (DAPI; dilactate; Invitrogen; 1:1000).

For immunocytochemistry staining of mixed glia cell culture, cells were fixed with 2% PFA for 30 min. Afterwards, cells were treated with 0.3% Triton-X-100 (Carl Rothe, Germany) in 10% normal goat serum (S26-100 ml, Merck KGaA, Germany) blocking buffer for 1 h. For microglia staining, we used primary monoclonal rat antibody against CD11b (MCA711; Bio-Rad/Serotec; 1:100). Astrocytes were stained with primary monoclonal mouse antibody against glial fibrillary acidic protein (GFAP, 173 011, Synaptic Systems; 1:200). BTK positive cells were identified by a polyclonal rabbit antibody (#8547, Cell signaling; 1:20). Primary antibodies were applied for 24 h at 4 °C. Anti-rat Cy3 (112-165-143, Jackson ImmunoResearch; 1:200), anti-mouse Alexa Fluor 488 (115-545-003, Jackson ImmunoResearch, 1:200), anti-rabbit Cy5 (111-175-144, Jackson ImmunoResearch, 1:200) were incubated at room temperature for 1 h. Nuclear staining was done by incubation with 4′,6-diamidino-2-phenylindole (DAPI, dilactate; Invitrogen; 1:1000) for 30 min. The slides were covered with fluorescence mounting medium (S3023, Dako, Hamburg, Germany), and captured using a digital camera (DP80, Olympus Europe GmbH) mounted by a fluorescence microscope (BX63, Olympus Europe GmbH).

### Isolation of adult murine microglia

Brains and spinal cords were isolated from mice upon perfusion with PBS and were dissociated to single cells according to manufactures protocol using a multi tissue dissociation kit (Miltenyi Biotec).

### Isolation of murine leukocytes

Single cell suspensions of murine lymphoid tissues were generated by gentle dissection and passing through 70 µm cell strainer (Greiner Bio-One). Murine B cells (purity > 95%) were isolated from spleens by MACS using anti-CD19 MicroBeads (Miltenyi Biotec).

### Generation of primary microglia and astrocytes

To generate primary microglia, brain cells of newborn to 2-day-old C57BL/6 mice were isolated enzymatically using 2.5% trypsin (Pan Biotech) and 0.4 mg DNAse I (Roche). First, a mixed glial cell culture was generated by seeding the cells in DMEM containing 10% fetal calf serum, 1% GlutaMax™, 100 U/ml penicillin, and 100 µg/ml streptomycin and cultivating them at 37 C and 5% CO_2_ until confluency was reached. To obtain enriched microglia cultures, cells were thereafter stimulated for 5 days with medium containing 30% conditioned L929 cell supernatant (DMEM, 30% L929 supernatant, 10% fetal calf serum, 100 U/ml penicillin, and 100 µg/ml streptomycin). Primary microglia were harvested by gentle shaking at 90 rpm for 30 min at 37 °C to separate microglial cells from other glia cell. Cultures usually contained > 95% microglial cells verified by flow cytometry. To obtain an enriched astrocyte culture, mixed glia cells were shaken off to remove other glial cells followed by negative MACS separation using a CD11b Biotin-labeled antibody (BD Bioscience), followed by an incubation with anti-Biotin magnetic microbeads (Miltenyi Biotech). Cultures usually contained > 95% astrocytes.

### Generation of human monocytes

PBMCs were isolated from human whole blood using the Ficoll-Paque density centrifugation methods. Monocytes were isolated by positive selection using the Miltenyi Pan monocyte isolation kit (130-096-537) according to manufacturer’s instructions. Monocytes were stimulated with 100 ng/ml recombinant human GM-CSF (PeproTech #300-03) for M1 differentiation and 50 ng/ml recombinant human M-CSF (PeproTech #300-25) for M2 differentiation, respectively and kept in culture in the presence or absence of evobrutinib for various time periods for gene expression experiments.

### THP-1 cell stimulation and western blot

THP-1 cells were washed with PBS and resuspended in DMEM (Gibco # 11966025). Cells were treated with 1 µM tool BTK inhibitor (MSC2498392) for 30 min at 37 ˚C. 100 ng/ml recombinant human GM-CSF (PeproTech #300-03) was added to the cells without washing and the following stimulation time course was started: 5, 15, 30 min. At the end of each timepoint, cells were washed twice with PBS (Gibco #10010023) and resuspended in lysis buffer (MSD-Tris lysis Buffer, #R60TX-3, Mesoscale). Lysates were processed for western blot analysis using Wes (proteinsimple, #SM-W004-1), according to manufacturer’s instructions. p-BTK and GAPDH antibodies were purchased from Cell Signaling Technologies (#5082S and #2118L, respectively).

### Cases, biopsy material and histology

Formalin-fixed, paraffin-embedded (FFPE) and cryoconserved brain tissue post mortem was taken. Two female individuals with MS were analyzed. The individual was diagnosed with Secondary progressive MS, age 58, male. The tissue was selected according to the presence of active and chronic active lesions, which was confirmed by histopathological examination. All sections were stained with haematoxylin eosin (HE) and Luxol fast blue/periodic-acid Schiff (LFB/PAS), HLA-DR+ and Bielschowsky silver impregnation according to standard procedures to assess cellularity, myelin, and axonal density, respectively. Immunohistochemistry (IHC) was performed with primary antibodies against BTK (abcam, Cambridge, UK, anti-rabbit, ab208937, 1:500) and ionized calcium-binding adaptor molecule 1 (Iba1, Merck, Darmstadt, Germany, MABN92, anti-mouse, 1:100) for visualization of microglia. Peroxidase and alkaline phosphatase conjugated secondary antibodies (Jackson Immuno Research, West Grove, USA, and DAKO, Santa Clara, USA) developed in diaminobenzidine (DAB, Merck, Darmstadt, Germany) for BTK and fast blue BB (Sigma, St. Louis, USA) were used for immunohistochemistry. Sections were scanned using the virtual slide microscope VS120 and visualized using the VS-ASW software (Olympus, Tokyo).

### ELISA

Production of, CCL2, IFN-γ, IL-17, TNF-α and GM-CSF was measured using ELISA MAX Standard Set kits (BioLegend), CCL3, CCL5 and CXCL10 production was measured employing ELISA MAX Standard Set kits (R&D Systems). Absorbance was measured at 450 nm with subtraction of a 540 nm reference wavelength on iMark microplate reader (Bio-Rad laboratories Inc.).

### Flow cytometry

Composition of murine immune cells was analyzed using the following antibodies: CD3 (145-2C11; BioLegend), CD19 (6D5; BioLegend), CD20 (SA275A11; BioLegend), CD11b (M1/70; BioLegend), CD11c (N418; BioLegend), CD45 (30-F11; BioLegend), Ly6C (HK1.4; BioLegend) and Ly6G (1A8: BioLegend). B cell maturation was analyzed using the following antibodies: CD19 (6D5; BioLegend), CD21 (7G6; BD Bioscience), CD23 (B3B4; BD Bioscience), CD93 (AA4.1; BioLegend), CD45R/B220 (RA3-6B2; BioLegend), IgD (11-26c.2a; BioLegend) and IgM (AF6-78; BD Bioscience). Monocyte, macrophage and microglia activation, differentiation and molecules involved in antigen presentation were determined using: CD40 (3/23; BD Bioscience), CD68 (FA-11; BioLegend), CD69 (H1.2F3; BioLegend), CD80 (16-10A1; BioLegend), CD86 (GL-1; BioLegend), MHCII (AF6-120.1; BioLegend) and PD-L1 (MIH5; eBioscience). Fc receptors were blocked using monoclonal antibody specific for CD16/ CD32 (93; BioLegend). Dead cells were stained with a fixable viability kit (BioLegend). Samples were acquired on a BD LSR Fortessa (BD Bioscience). All data evaluation was performed using FlowJo software (FlowJo LLC, Ashland, USA).

Intracellular proteins were analyzed using the BD PhosFlow protocol and analyzed using the following antibodies: BTK (53/BTK; BD Bioscience), pBTK (N35-86, BD Bioscience), iNOS (W16030C, Biolegend) Arg1 (A1exF5, eBioscience).

### Quantitative PCR

RNA was isolated using the RNeasy mini kit (Qiagen) and transcribed into cDNA using the QuantiNova Reverse Transcription kit (Qiagen). Quantitative (q) PCR was performed using 500 nM Primer and qPCRBIO SyGreen in a total volume of 10 µl on a QuantStudio 7. Primers specific for BTK (forward: 5′-GGTGGAGAGCACGAGATAAA-3′, reverse: 5′CCGAGTCATGTGTTTGGAATAC-3′) and B2M (forward 5′- CGGCCTGTATGCTATCCAGA-3′, reverse: 5′ GGGTGAATTCAGTGTGAGCC-3′) were purchased from Eurofins Genomics. Primer for human IL-10 (Hs00961622_m1) and IL-1ß (Hs00174097_m1) were purchased from ThermoFisher. qPCR was performed at 95 °C desaturating and 70 °C annealing temperature for 30 s and 40 cycles with subsequent melt-curve analysis. Primer specificity was validated by product size using a 2% Agarose gel containing GelRed and UV-light illumination. Samples were analyzed in duplicate or triplicate and considered valid when cycle threshold (C_t_) < 35 and standard deviation of C_t_ < 0.5. The relative expression was determined in comparison to the control-treated group.

### Analysis of Evobrutinib by LC–MS/MS

C57BL/6 J mice were treated orally, daily for indicated days with evobrutinib or vehicle control. 30 min after the final dose, all animals were perfused with PBS and brains were collected and immediately snap-frozen using liquid nitrogen. The concentrations of evobrutinib in brain samples was determined by liquid chromatography with tandem mass spectrometry (LC–MS/MS).

Stock solution of evobrutinib and D8-Imatinib (Alsachim) were prepared in following concentrations and stored at −20 °C. Calibrators and quality control samples have been prepared in brain matrix following concentration and stored at −20 °C until use.

Calibrators, qc-samples as well as mouse brain samples were prepared pipetting 90 µl sample, 10 µl internal standard and 100 µl Acetonitrile (Carl Roth). Samples were vortexed for 2 min followed centrifugation at 12,000 rpm for 15 min and 4 °C. Supernatant was transferred into a hplc vial (Waters™) and cooled until analysis. 10 µl of supernatant was injected into LC–MS/MS system for analysis.

The LCMS system composed of a Nexera (TM)X2 UHPLC and a LC–MS-8060 (TM) LC–MS/MS (Shimadzu Corporation, Kyoto Japan), was used for determination and quantification of evobrutinib. Direct injection with a sharp short gradient was used for chromatographic separation. Separation was performed on a velox C18 2.1 × 50 mm 1.7 µm column (Shimadzu Corporation Kyoto Japan) at an oven temperature of 30 °C. Mobile phase A: 0.1% Ammoniumformiat, 0.02% formic acid pH3.7 and Mobile Phase B: methanol containing 0.1% Ammoniumformiat, 0.02% formic acid were used running following gradient with a flowrate of 0.6 mL/min: Gradient 0 min 1%B, 0.2 min 20%B, 2 min 95%B, 2.4 min 95%B, 2.5 min 1%B; total run time 3.5 min. After 1 min, the waste valve was switched to mass spectrometer. For ionization and fragmentation the following ms-conditions were used: interface temp 300 °C DL 250 °C, Heat block temp 400 °C, argon was used as reactant gas with 270 psi. Ion current of the optimized MRM transitions for evobrutinib 430.2/98.1; 430.2/279.1 (31%) 430.2/152.1 (15%), and internal standard D-8 imatinib 502.2/394.2; 502.2/225.3 (22%); 502.2 > 247.1 (18%) were measured with 5 ms dwell time. All validations results were within the acceptance criteria. Calibration was linear from 0.1 to 1000.0 µg/L (*r*^2^ = 0.9998487). QC and calibrant accuracy were within 85–115%), within run of qc-samples (*n* = 3) result in following cv for given concentrations: 0.1 µg/L (cv 17.7%); 1.0 µg/L (cv 1.48%); 10.0 µg/L (cv 1.48%) and 100 µg/L (1.75%). Between run qc results (*n* = 5) were at 1.0 µg/L (cv 1.48%); 10.0 µg/L (cv 1.48%) and 100 µg/L (1.75%). Samples were measured in duplicates. All results had a CV less than 5%. LCMS repeatability (area, area ratio and ion ratio RSD below 15%), LLOQ confirmation (signal-to-noise ratio above 10 and area RSD below 15%). Carryover was less than 0.5%.

Plasma (Fig. 2b and Supplementary Fig. 2b) and brain samples (Supplementary Fig. 2b) were analyzed by LC–MS/MS for MSC2364447 concentrations. MSC2364447 stock standard solutions containing 2 to 2500 ng/mL of MSC2364447 were made up in methanol through serial dilutions from a 2 mg/ml DMSO stock solution and pre-pared for analysis in a 96-well plate with the following additions: 25 µl plasma (blank), 25 µl standard, 25 µl MSC1936369 (internal standard; 5 µg/ml in methanol), 100 µl acetonitrile and 50 µl of water. Samples from dosed mice were prepared similarly in a 96-well plate with the following additions: 25 µl plasma (unknown), 25 µl methanol, 25 µl MSC1936369 (internal standard; 5 µg/ml in methanol), 100 µl acetonitrile and 50 µl water. Plates were mixed thoroughly and filtered through Captiva filter plates (0.45 um pore). The filtrate was analyzed by liquid chromatography-tandem mass spectrometry (LC–MS/MS) and concentrations of MSC2364447 were calculated using the standard curve. Samples having concentrations lower than 2 ng/ml were reanalyzed using a standard curve prepared from stock standard solutions containing 0.1 to 10 ng/ml of MSC2364447 in the same manner. The lower level of quantification (LLOQ) in plasma was 0.1 ng/ml.

### BTK occupancy assay

BTK occupancy in plasma: Briefly, 80 µl of blood was combined with 800 µl of red cell lysis buffer and lysis was allowed to occur for 5 min at room temperature. The white blood cells were pelleted by centrifugation at 600 × g, washed once with 1 ml of red cell lysis buffer and resuspended in 1 ml of RPMI 1640. The biotinylated probe, MSC2527393, was added to a final concentration of 1 µM and the cells were maintained at 37 °C in 5% CO_2_ in an incubator for 1 h. After the incubation, the cells were pelleted and 80 µl of MPER lysis buffer was added after aspiration of the supernatant. Samples were then frozen at -80 °C for later testing in the MSD assay.

BTK occupancy in brain: After perfusion, one half of a mouse brain was placed into a Miltenyi C-tube containing 3 ml of RPMI 1640 and homogenized using the Miltenyi OctoMacs on program mSpleen 01_01. Two cycles were performed to ensure complete homogenization. The tubes were subsequently centrifuged 2 min at 500 × g and the supernatant was discarded. The homogenate was resuspended in 4 ml of RPMI 1640 and 1 ml was transferred to a 1.5 ml Eppendorf tube and centrifuged 5 min at 400 × g. The supernatant was discarded and the homogenate was resuspended in RPMI 1640 containing 1 µM of the biotinylated probe MSC2527393. The mixture was incubated for 1 h at 37 °C in 5% CO2 in an incubator. After incubation, the homogenate was centrifuged 5 min at 400 × gg, the supernatant was discarded and the cell pellet was resuspended in 250 µl of MPER lysis buffer. After incubation on ice for 10 min, the samples were frozen for later analysis in the MSD assay.

MSD assay: for occupancy measures, experimental samples were analyzed for biotinylated probe binding to BTK using a streptavidin capture MSD assay. Briefly, MSD 96 well plates coated with streptavidin were blocked by incubation with casein blocking buffer for 1 h at room temperature. After blocking, probe-treated blood or brain lysates diluted in blocking buffer were added to wells of the plate and incubated for 2 h at room temperature with gentle shaking. The plate was then washed and incubated with a rabbit anti-BTK antibody for 1.5 h followed by incubation with an anti-rabbit IgG SULFO-tagged antibody with gentle shaking for both incubations. After washing, 2X MSD Read Buffer was added and the plate was read using the MSD Sector 600 Imager and the results analyzed using the MSD Discovery Workbench software program. A standard curve employing recombinant BTK previously treated with biotinylated probe in vitro was used for quantitation. Curve fitting of the standards was performed using a four parameter fit.

### Statistical analysis

Statistics were calculated using GraphPad Prism 6. For the analysis of in vitro experiments, Gauss distribution was assumed. The respective statistical comparisons used are indicated in the figure legends.

### Supplementary Information

Below is the link to the electronic supplementary material.

Supplementary file1 (TIF 47046 kb) BTK is upregulated in infiltrating cells in the spleen under CNS inflammation. a-c) C57BL/6 mice were immunized with MOG peptide 35-55. Immune cells (Lymphocytes: CD45+CD11b-, monos/macrophages: CD11b+CD45hiLy6Clow, mon-os/macrophages: CD11b+CD45hiLy6Chi, neutrophils: CD11b+CD45hiLy6C+Ly6G+) were isolated a) from the spleen b) brain and c) spinal cord and BTK expression was analysed by flow cytometry. Data are shown as mean fluorescence intensity (MFI, n=4). d) Microglia (CD11b+CD45intLy6C-Ly6G-ACSAII-O4-), astrocytes (CD11b-ACSAII+O4-) and oligodendrocytes (CD11b-ACSAII-O4+) were isolated from the brain and spinal cord from C57BL/6 mice and BTK expression was analysed by flow cytometry. e) Brain biopsy of chronically active (smouldering) MS lesion co-stained for BTK expression in Olig2 positive cells. f) Brain biopsy of chronically active (smouldering) MS lesion. I+II: HE stained section with sharply demarcated lesion (bottom of I) displaying a lesion rim consisting of activated microglial cells with elongated nuclei (II: magnification of area depicted in I, arrows point at microglial cells). Note the almost complete absence of foamy macrophages as well as perivascular lymphocytic cuffs. III+V: Double immunolabeling against proteolipid protein (PLP) visualizing myelin loss at the lesion center (bottom of III) and against HLA immunopositive microglial cells (V: magnification of area depicted in III, arrows point at microglial cells). Note the abundance of microglial cells at the lesion rim. The mean ± standard error of the mean is indicated in all graphs. Data sets are representative of at least two independent experiments. Asterisks indicate significant differences calculated using a-c) unpaired two-tailed t-test (*P ≤ 0.05, **P ≤ 0.01, ***P ≤ 0.001)

Supplementary file2 (TIF 90473 kb) Evobrutinib inhibition in the acute EAE and passive EAE. a-c) EAE was induced in SJL/J mice using an emulsification of PLP139-151 (100 mg/mouse) peptide and IFA (supplement-ed with 2 mg/mL Mycobacterium tuberculosis) on day 0. Mice also received a pertussis toxin (60 ng/mouse) injection (i.p.) on day 0 and 2. Treatment with 3 mg/kg Evobrutinib or vehicle control was initiated at the Day Post Immunization 0 (DPI 0) and continued daily (n=5-18). b) For determination of circulat-ing plasma evobrutinib concentrations, plasma at 2 h after last dose at day 14 was prepared, while brain was collected after perfusion and frozen at the same timepoints. Evobrutinib exposure levels in plasma and brain samples were analyzed by LC-MS/MS (n=5). c) BTK occupancy was measured in the blood and the brain, samples were collected 2 h after last dose (n=5). d-m) C57BL/6J mice were treated daily with 10mg/kg evobrutinib or vehicle control started 3 days prior to passive EAE induction with pathogenic T cells. d) Schematic overview of experimental setup. e) Evobrutinib concentration in brain homogenates isolated from mice at day 12 after T cell transfer. Tissue was collected 30 minutes after final dose. f) Com-position of encephalitogenic T cells after in vitro stimulation before adoptive transfer. g) Phenotype of encephalitogenic T cells were analysed by flow cytometry before adoptive transfer. h, i) B cells were isolated from the spleen on peak of disease (day 12) and d) maturation and e) activation were analysed by flow cytometry (n=9-10). j) CD11b+ cells were iso-lated from the spleen on peak of disease (day 12) and activation was analysed by flow cytometry (n=9-10). . k-l) Infiltrating lymphocytes were isolated from the brain (k) and spinal cord (l) on peak of disease (day 12) and activation were analysed by flow cytometry (n=9-10). m) Gating strategy of microglia isolated from the CNS (CD11b+CD45lowLy6C-Ly6G-). The mean ± standard error of the mean is indicated in all graphs. Data sets are representative from at least two independent experi-ments. Asterisks indicate significant differences calculated using one-way analysis of variance corrected by Holm-Sidak (*P ≤ 0.05, **P ≤ 0.01, ***P ≤ 0.001, ****P ≤0.0001)

Supplementary file3 (TIF 56509 kb) BTK inhibition changes microglia state in an inflammatory milieu. Primary microglia were either left unstimulated, treated with indicated concentrations of evobrutinib or DMSO control followed by stimulation a-d) with 0.5 ng/ml LPS for 18h or e-h) stimulated with 10 ng/ml IFNγ+IL1-β for 18h. a-h) Changes in the expression of disease-associated microglial markers were analysed by flow cytometry. Data are normalized to vehicle control and are shown as mean fluorescence intensity, (MFI, n=4-8, pooled from at least 3 independent experiments). i-k) Primary astrocytes were either left unstimulated, treat-ed with indicated concentrations of evobrutinib or DMSO control followed by stimulation with a combination of 0.1 ng/ml IFNγ+IL1-β for 18h. Cytokine concentrations were determined by ELISA (n=4 wells/condition. Mean ± standard error of the mean is indicated in all graphs. Asterisks indicate significant differences calculated using a-h) one-way analysis of variance corrected by Holm-Sidak (*P ≤ 0.05, **P ≤ 0.01, ***P ≤ 0.001, ****P ≤0.0001)

Supplementary file4 (TIF 12877 kb) Evobrutinib inhibits phosphorylation of BTK THP-1 monocytes. THP1 cells were treated with the evobrutinib tool compound (cmp) for 30 minutes followed by stimulation with GM-CSF (100 ng/mL) for indicated time. Cells were lysed subsequently and BTK phosphorylation at Y223 was analyzed by western blot. Phosphorylated BTK signal was normalized to GAPDH for quantification (n=1)

Supplementary file5 (TIF 39797 kb) Inhibition of BTK during demyelination in in the cuprizone mouse model. C57BL/6J mice were treated daily with 10mg/kg evobrutinib or vehicle control started 3 days prior to simultaneously cuprizone diet for 5 weeks (b-k) or a) with withdrawal of the cuprizone diet on day 35. a, b) body weight (n =9-10). c) Myelinated areas in the corpus callosum (CC) were assessed by luxol fast blue/periodic acid-Schiff (LFB/PAS) staining and are shown as percentage of myelinated CC in relation to the total CC. d, h) Anti-myelin proteolipid protein (PLP) staining and are shown as percentage of myelinated CC in relation to the total CC (d) or scoring of the cortex area (h). Immunostaining of e, i) oligodendrocyte transcription factor 2 (Olig2), number of cells/mm2 per group. f, j) Microglia (Iba1) and g, k) astrocytes (GFAP), fluorescence intensity in CC/cortex in relation to the total CC or cortical area. l, m) The red marked area of the CC and cortex which were analysed. c-k) Mean ± standard error of the mean (SEM). Data are normalized to vehicle and pooled from at least two independent experiments (n=7-11). Asterisks indicate significant difference calculated using the unpaired two-tailed t-test (*P ≤ 0.05, **P ≤ 0.01, ***P ≤ 0.001)

## Data Availability

All data associated with this study are included in the main figures or the supplementary figures/tables (online resource).
