# Supplementary figures and images for: BTK inhibition limits microglia-perpetuated CNS inflammation and promotes myelin repair
Source: Acta Neuropathol. 2024 Apr 24;147(1):75. doi: 10.1007/s00401-024-02730-0 (PMC11043151; doi:10.1007/s00401-024-02730-0)

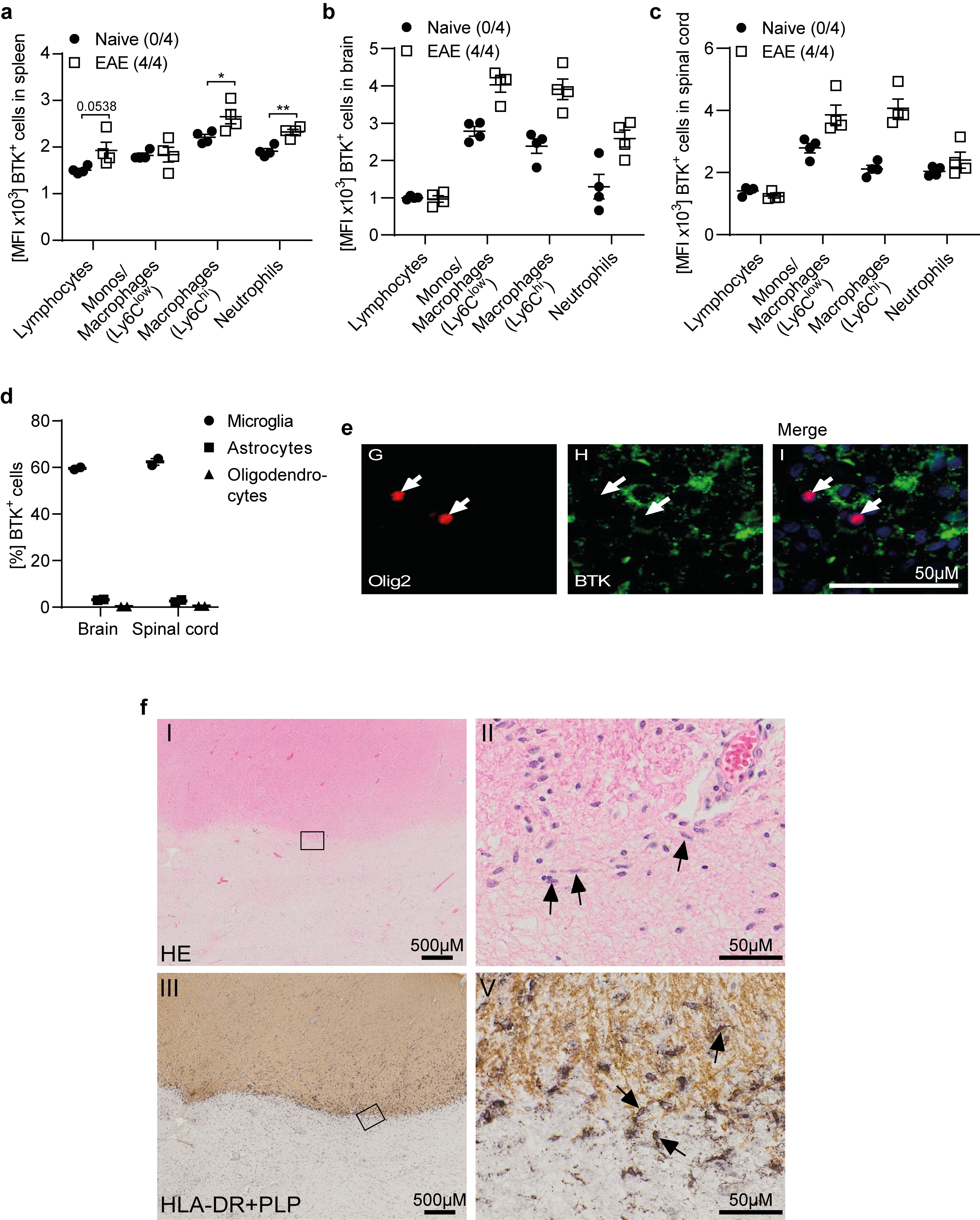

Supplement: Supplementary file 1 — Supplementary file1 (TIF 47046 kb) BTK is upregulated in infiltrating cells in the spleen under CNS inflammation. a-c) C57BL/6 mice were immunized with MOG peptide 35-55. Immune cells (Lymphocytes: CD45+CD11b-, monos/macrophages: CD11b+CD45hiLy6Clow, mon-os/macrophages: CD11b+CD45hiLy6Chi, neutrophils: CD11b+CD45hiLy6C+Ly6G+) were isolated a) from the spleen b) brain and c) spinal cord and BTK expression was analysed by flow cytometry. Data are shown as mean fluorescence intensity (MFI, n=4). d) Microglia (CD11b+CD45intLy6C-Ly6G-ACSAII-O4-), astrocytes (CD11b-ACSAII+O4-) and oligodendrocytes (CD11b-ACSAII-O4+) were isolated from the brain and spinal cord from C57BL/6 mice and BTK expression was analysed by flow cytometry. e) Brain biopsy of chronically active (smouldering) MS lesion co-stained for BTK expression in Olig2 positive cells. f) Brain biopsy of chronically active (smouldering) MS lesion. I+II: HE stained section with sharply demarcated lesion (bottom of I) displaying a lesion rim consisting of activated microglial cells with elongated nuclei (II: magnification of area depicted in I, arrows point at microglial cells). Note the almost complete absence of foamy macrophages as well as perivascular lymphocytic cuffs. III+V: Double immunolabeling against proteolipid protein (PLP) visualizing myelin loss at the lesion center (bottom of III) and against HLA immunopositive microglial cells (V: magnification of area depicted in III, arrows point at microglial cells). Note the abundance of microglial cells at the lesion rim. The mean ± standard error of the mean is indicated in all graphs. Data sets are representative of at least two independent experiments. Asterisks indicate significant differences calculated using a-c) unpaired two-tailed t-test (*P ≤ 0.05, **P ≤ 0.01, ***P ≤ 0.001) [file 401_2024_2730_MOESM1_ESM.tif]

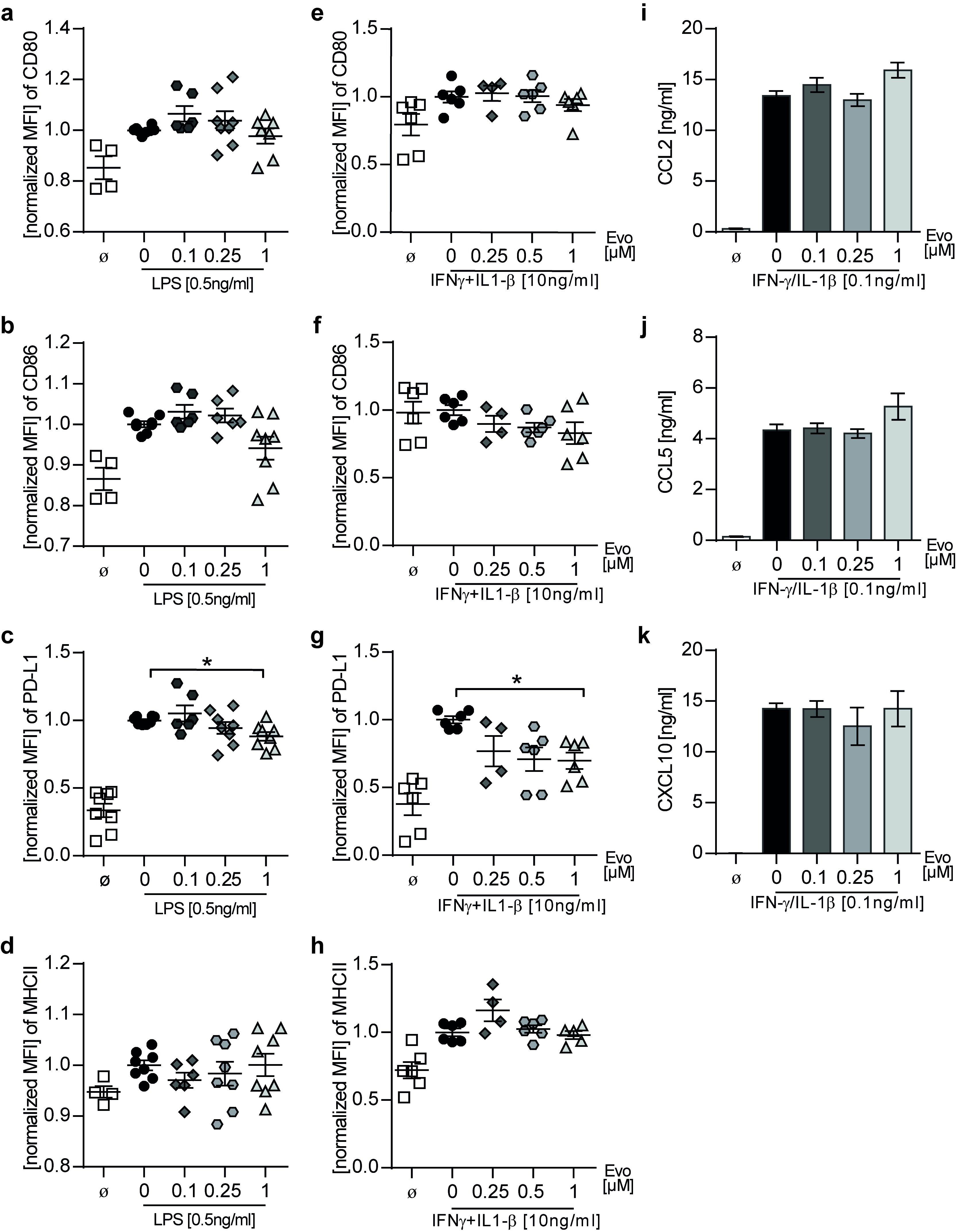

Supplement: Supplementary file 3 — Supplementary file3 (TIF 56509 kb) BTK inhibition changes microglia state in an inflammatory milieu. Primary microglia were either left unstimulated, treated with indicated concentrations of evobrutinib or DMSO control followed by stimulation a-d) with 0.5 ng/ml LPS for 18h or e-h) stimulated with 10 ng/ml IFNγ+IL1-β for 18h. a-h) Changes in the expression of disease-associated microglial markers were analysed by flow cytometry. Data are normalized to vehicle control and are shown as mean fluorescence intensity, (MFI, n=4-8, pooled from at least 3 independent experiments). i-k) Primary astrocytes were either left unstimulated, treat-ed with indicated concentrations of evobrutinib or DMSO control followed by stimulation with a combination of 0.1 ng/ml IFNγ+IL1-β for 18h. Cytokine concentrations were determined by ELISA (n=4 wells/condition. Mean ± standard error of the mean is indicated in all graphs. Asterisks indicate significant differences calculated using a-h) one-way analysis of variance corrected by Holm-Sidak (*P ≤ 0.05, **P ≤ 0.01, ***P ≤ 0.001, ****P ≤0.0001) [file 401_2024_2730_MOESM3_ESM.tif]

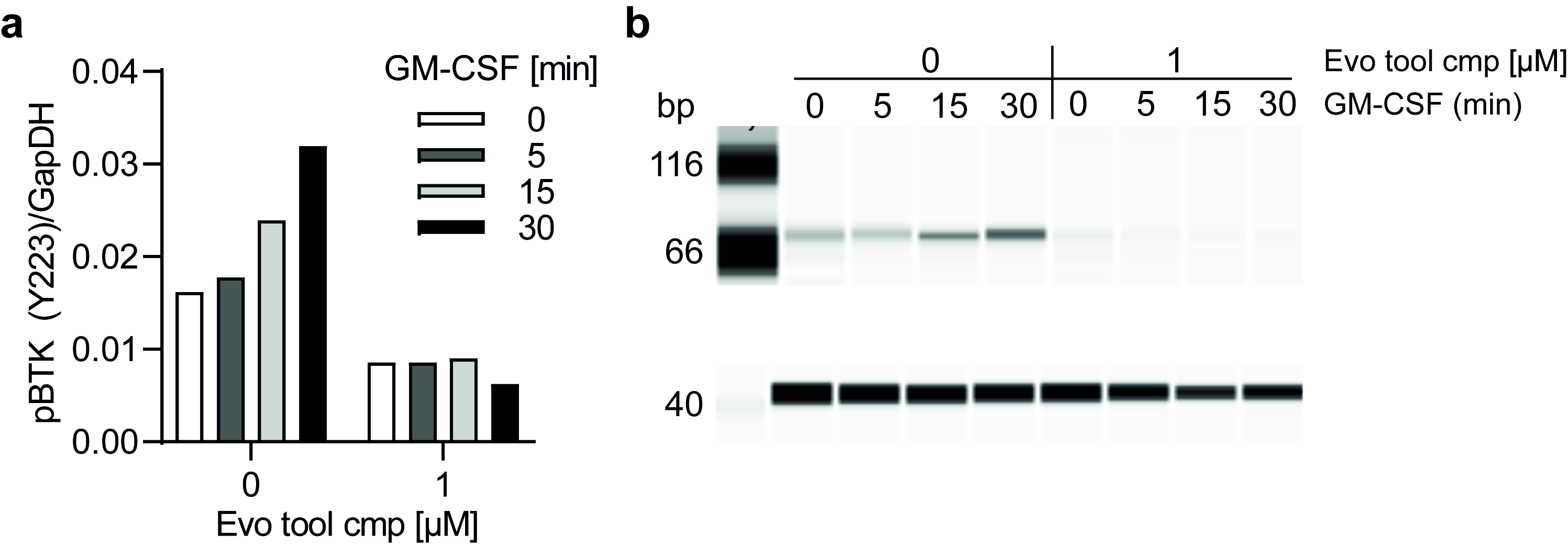

Supplement: Supplementary file 4 — Supplementary file4 (TIF 12877 kb) Evobrutinib inhibits phosphorylation of BTK THP-1 monocytes. THP1 cells were treated with the evobrutinib tool compound (cmp) for 30 minutes followed by stimulation with GM-CSF (100 ng/mL) for indicated time. Cells were lysed subsequently and BTK phosphorylation at Y223 was analyzed by western blot. Phosphorylated BTK signal was normalized to GAPDH for quantification (n=1) [file 401_2024_2730_MOESM4_ESM.tif]

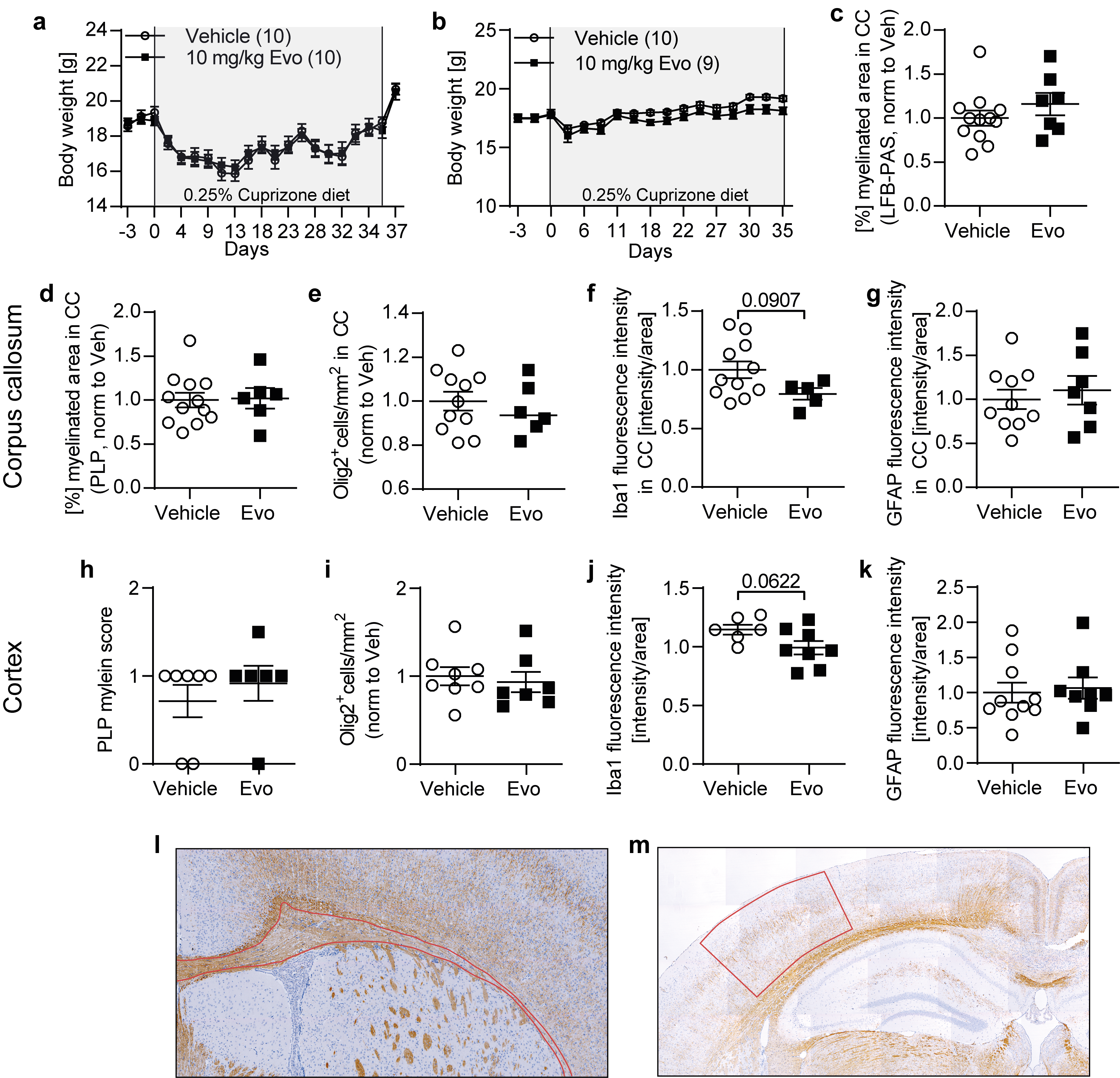

Supplement: Supplementary file 5 — Supplementary file5 (TIF 39797 kb) Inhibition of BTK during demyelination in in the cuprizone mouse model. C57BL/6J mice were treated daily with 10mg/kg evobrutinib or vehicle control started 3 days prior to simultaneously cuprizone diet for 5 weeks (b-k) or a) with withdrawal of the cuprizone diet on day 35. a, b) body weight (n =9-10). c) Myelinated areas in the corpus callosum (CC) were assessed by luxol fast blue/periodic acid-Schiff (LFB/PAS) staining and are shown as percentage of myelinated CC in relation to the total CC. d, h) Anti-myelin proteolipid protein (PLP) staining and are shown as percentage of myelinated CC in relation to the total CC (d) or scoring of the cortex area (h). Immunostaining of e, i) oligodendrocyte transcription factor 2 (Olig2), number of cells/mm2 per group. f, j) Microglia (Iba1) and g, k) astrocytes (GFAP), fluorescence intensity in CC/cortex in relation to the total CC or cortical area. l, m) The red marked area of the CC and cortex which were analysed. c-k) Mean ± standard error of the mean (SEM). Data are normalized to vehicle and pooled from at least two independent experiments (n=7-11). Asterisks indicate significant difference calculated using the unpaired two-tailed t-test (*P ≤ 0.05, **P ≤ 0.01, ***P ≤ 0.001) [file 401_2024_2730_MOESM5_ESM.tif]
